# Significance of Five-Membered Heterocycles in Human Histone Deacetylase Inhibitors

**DOI:** 10.3390/molecules28155686

**Published:** 2023-07-27

**Authors:** Anton Frühauf, Martin Behringer, Franz-Josef Meyer-Almes

**Affiliations:** Department of Chemical Engineering and Biotechnology, University of Applied Sciences, Haardtring 100, 64295 Darmstadt, Germany; anton.fruehauf@h-da.de (A.F.); martin.behringer@h-da.de (M.B.)

**Keywords:** histone deacetylase inhibitors, drug development, drug design, active pharmaceutical ingredient, active substance optimization

## Abstract

Five-membered heteroaromatic rings, in particular, have gained prominence in medicinal chemistry as they offer enhanced metabolic stability, solubility and bioavailability, crucial factors in developing effective drugs. The unique physicochemical properties and biological effects of five-membered heterocycles have positioned them as key structural motifs in numerous clinically effective drugs. Hence, the exploration of five-ring heterocycles remains an important research area in medicinal chemistry, with the aim of discovering new therapeutic agents for various diseases. This review addresses the incorporation of heteroatoms such as nitrogen, oxygen and sulfur into the aromatic ring of these heterocyclic compounds, enhancing their polarity and facilitating both aromatic stacking interactions and the formation of hydrogen bonds. Histone deacetylases are present in numerous multiprotein complexes within the epigenetic machinery and play a central role in various cellular processes. They have emerged as important targets for cancer, neurodegenerative diseases and other therapeutic indications. In histone deacetylase inhibitors (HDACi’s), five-ring heterocycles perform various functions as a zinc-binding group, a linker or head group, contributing to binding activity and selective recognition. This review focuses on providing an up-to-date overview of the different five-membered heterocycles utilized in HDACi motifs, highlighting their biological properties. It summarizes relevant publications from the past decade, offering insights into the recent advancements in this field of research.

## 1. Introduction

**Figure 1:**
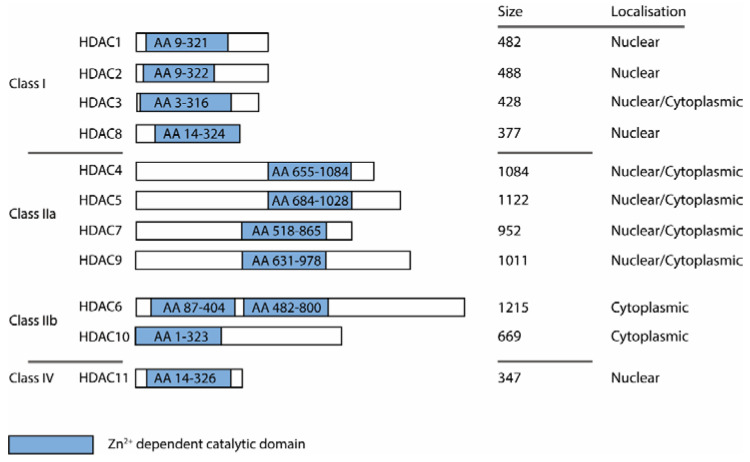
Schematic classification of HDAC isozymes into class I, IIa, IIb and IV. Indicated are catalytic domain size (blue box), protein length (size) and localization.

Heterocyclic compounds are a class of organic compounds that contain at least one atom other than carbon in the ring structure. The presence of heteroatoms such as nitrogen, oxygen and sulfur in the aromatic ring increases the polarity and offers the possibility of hydrogen bonds that can improve the binding affinity and selectivity of the drug at the target site. Heteroaromatic rings are considered preferred structures due to their ability to replace common motives in medicinal chemistry, enabling an improvement of metabolic stability, solubility and bioavailability, which are essential elements for the development of effective drugs [1,2,3]. These properties can lead to the modulation of different biochemical metabolic pathways, resulting in therapeutic effects. Among active compounds with aromatic groups, five-membered heterocyclic compounds are of great importance in the field of pharmaceutical and medicinal chemistry. Due to their unique physicochemical properties and biological effects, these compounds are used as key structural motifs in many clinically effective drugs. Therefore, the development and synthesis of novel five-membered heterocycles, remains an important research area in medicinal chemistry, with the aim of finding new drugs for the treatment of various diseases. Recent advances in the development of histone deacetylase (HDAC) inhibitors incorporating five-membered heterocyclic scaffolds emphasized the importance of heterocycles as valuable scaffolds in this area of research [4,5]. HDACs are encountered in many multiprotein complexes in the epigenetic apparatus, are involved in many cellular processes and are established targets for cancer, neurodegenerative diseases and other indication areas [6,7] with five clinically approved drugs to date [8,9,10,11,12]. The HDAC family is composed of 18 HDAC isozymes, which can be categorized as zinc or NAD+-dependent, whereas the latter are referred to as sirtuins (SIRT1-7) and will be excluded in this review [13,14]. The zinc-dependent HDAC isozymes can be grouped into four classes and vary in their size and localization as indicated in Figure 1.

**Figure 2:**
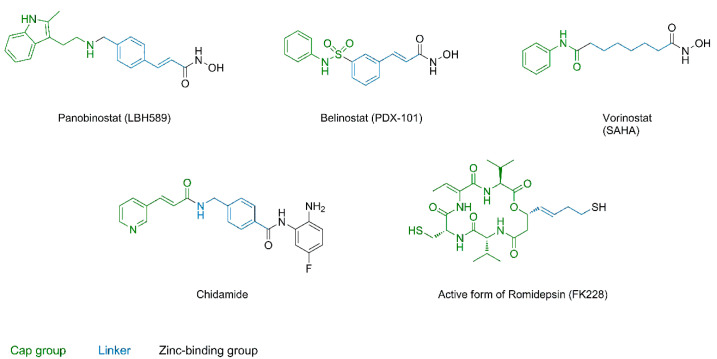
Approved HDAC inhibitors with indicated structural features. Cap group, linker and ZBG are labelled in green, blue and black, respectively.

**Figure 3:**
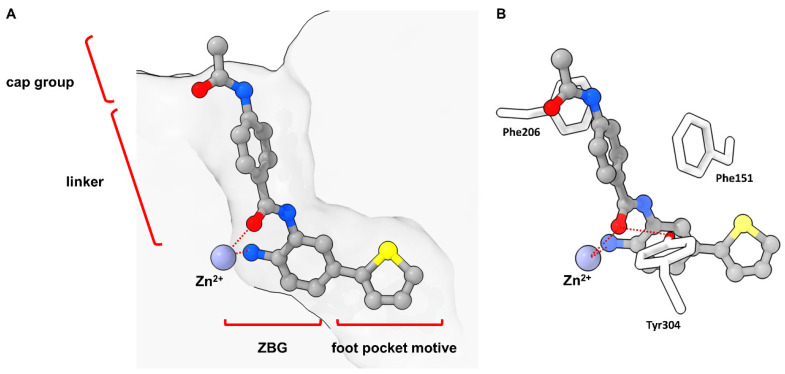
Exemplary binding pose of a benzamide HDACi with indicated structural features inside a HDAC2 binding pocket (PDB-ID: 4LY1, 1.57 Å) [15] illustrated using the UCSF ChimeraX visualization software (version ChimeraX-1.4) [16,17]. The inhibitor and the amino acid side chains are displayed in the ball and stick or the stick style and are colored by element or heteroatom, respectively. (**A**) Clipped binding pocket indicating the canonical binding pocket occupied by the linker and ZBG motive and the acetate release channel occupied by the foot pocket motive. The zinc ion is shown as bluish sphere and interacts with the ZBG group via indicated dotted red lines. (**B**) Most prominent residues and interactions in a typical class I binding site. The greatest energy contribution is provided by the interaction between the ZBG group and the zinc ion as well as hydrogen bond stabilization between Tyr304 and the carbonyl functionality of the HDACi, indicated as dotted red lines. The substrate tunnel is composed of hydrophobic residues of which Phe151 and Phe206 can be exploited for π-stacking interactions.

The structure of typical HDAC inhibitors (HDACis) can be simply illustrated consisting of three parts shown in Figure 2 and Figure 3: the cap moiety, which is responsible for protein surface interactions, the linker providing the optimal spacing as well as establishing favorable interactions in the substrate-binding tunnel, and the zinc binding group (ZBG) responsible for most of the binding affinity and isoform selectivity by establishing interactions in the active site and the foot pocket.

The aim of this review is to provide an overview of the different five-membered heterocycles in HDACi motifs and their biological properties. In particular, this will summarize publications from the last 10 years to provide an up-to-date overview. HDACis containing five-membered heterocycles are divided into two subchapters according to the localization of the heterocycle scaffold and its interaction with the target enzyme. Inhibitors in chapter 2 contain five-ring heterocycles that bind the catalytic zinc ion in the active site or interact with the foot pocket of certain HDAC isozymes. HDACis in chapter 3 contain five-ring heterocycles integrated either in the linker or in the cap group, allowing diverse molecular interactions with the protein surface.

## 2. ZBG Scaffolds

### 2.1. 1,2,4-Oxadiazoles

**Figure 4:**
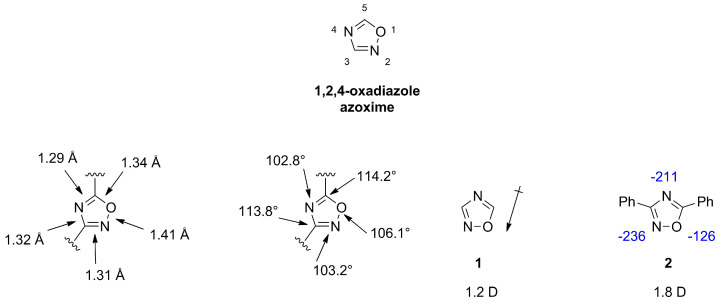
Schematic illustration of bond lengths (Å), bond angles (°), dipole moments (D) and calculated hydrogen bond acceptor strengths in kcal/mol indicated in blue.

1,2,4-oxadiazoles, also referred to as azoximes show diverse biological activities and are frequently employed in medicinal chemistry as hydrolysis resistant bioisosteric replacements for ester or amide functionalities [3,18]. The scaffold is a weak base and can act only as hydrogen bond acceptor. X-ray measurements of azoximes show a planar ring and suggest a double bond character for both C-N distances, which is in agreement with a lower value of 39 for the aromatic character compared to furan with a value of 43, as determined by the bird index [18,19]. The dipole moment for unsubstituted **1** (1.2 D) or substituted **2** (1.8 D) azoximes is lower compared to other oxadiazoles (Figure 4) [2,20].

**Figure 5:**
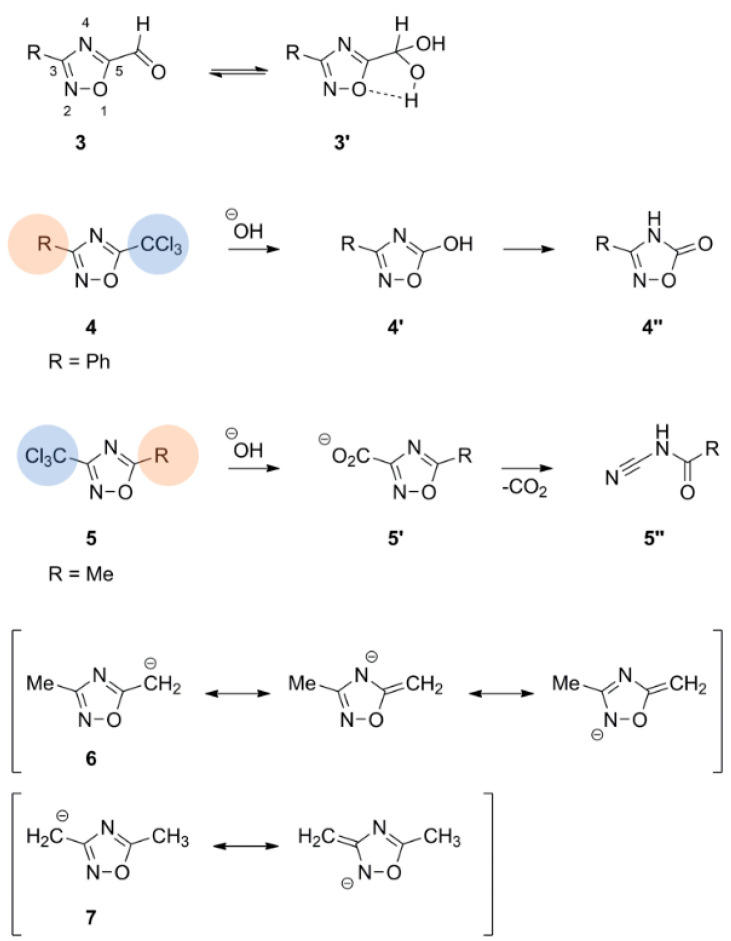
Properties and reactivities of 1,2,4-oxadiazole derivatives. The electron-withdrawing nature of azoximes is indicated by diol formation of **3**. C3 and C5 reactivity is illustrated with **4** and **5** in the presence of electron withdrawing groups and can be rationalized with mesomeric structures of **6** and **7**.

Disubstituted azoximes are stable and can be heated in conc. sulfuric acid or recrystallized from nitric acid and show only slow decomposition at 250 °C. Mono- or unsubstituted azoximes are thermally and hydrolytically less stable [18,20]. Azoximes are rather inert against electrophilic attacks as halogenation, nitration and Friedel Crafts acylation or alkylation do not occur [20]. Formation of a diol moiety in **3** indicates the electron withdrawing nature of this scaffold [20]. Reactivities observed for C3 and C5 substitutions in examples **4** and **5** can be rationalized by mesomeric structures of **6** and **7**. Nucleophilic attack on C5 but not C3 can displace a leaving group (Figure 5) [20].

Trifluoro-1,2,4-oxadiazole (TFMO) HDACi scaffolds were published by Lobera et al. in 2013 and exhibit outstanding class IIa selectivity [4]. This finding was built upon by future publications employing TFMO HDACis in various studies, which are reported below. Class IIa selectivity is of high interest in various cases like Huntington disease, where an overexpression of HDAC4, a class IIa representative, was observed. These findings indicate the TFMO moiety to be a valuable probe and a potential candidate for clinical studies.

**Figure 6:**
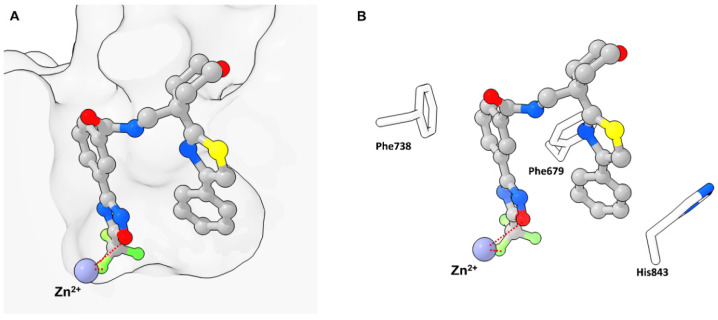
Representation of **TMP269** bound to HDAC7 (PDB-ID: 3ZNR, 2.35 Å) illustrated using the UCSF ChimeraX visualization software (version ChimeraX-1.4) [16,17]. The inhibitor and the amino acid side chains are displayed in the ball and stick or the stick style and are colored by element or heteroatom, respectively. (**A**) Notable is the larger binding site of class IIa HDACs compared to other HDAC classes. Possible interactions between **TMP269** and the zinc ion are indicated as dotted red lines. (**B**) Most prominent residues and interactions with **TMP269**. The distal “cap”-phenyl moiety of **TMP269** occupies the foot pocket and displaces His843 with an edge-to-face orientation towards Phe679.

In 2013 Lobera et al. published a novel class IIa selective HDACi with a trifluoromethyl-1,2,4-oxadiazole (TFMO) motive as zinc binding group (ZBG) identified in a high-throughput screening (HTS) and used it to study gene regulation in phytohemagglutinin (PHA)-activated human peripheral blood mononuclear cells (PBMCs) [4]. The obtained crystal structure (PDB-ID: 3ZNR, 2.35 Å) of **TMP269** bound to HDAC7 showed a U-shaped conformation of the inhibitor (Figure 6). The TFMO moiety occupies the active site and interacts with the zinc ion via the fluorine and oxygen atoms with distances of 2.7 Å and 3.0 Å, respectively. Since hydroxamates have shorter interaction distances of ~2 Å, the authors concluded the TFMO interactions to be of weak electrostatic nature. The selectivity was rationalized by a larger active site in class IIa HDACs, which readily accommodates the TFMO moiety. Interactions of the lipophilic tail in the hydrophobic foot pocket led to a displacement of His843 and an edge-to-face orientation of the phenyl group to Phe679.

**Figure 7:**
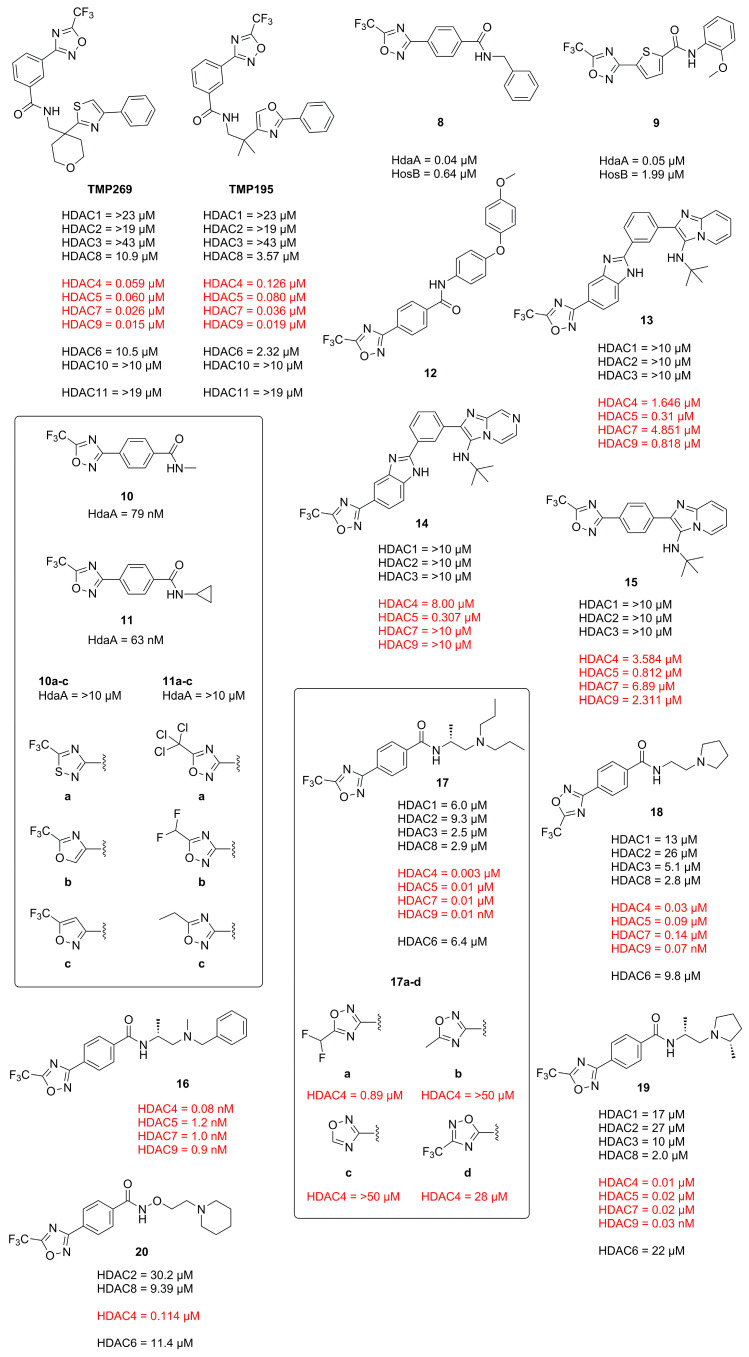
Summary of compounds mentioned in Section 2.1. SAR studies of the TFMO group are marked by a box. Inhibitory activity against class IIa HDACs is highlighted in red.

Compound **TMP195** (Figure 7) was chosen as representative with an activity of 300 nM in cell-based class IIa HDAC assays and up- or downregulated only 16 and 60 genes by a factor of ≥1.4, respectively. Comparatively the pan-HDACi vorinostat up- or downregulated 1853 and 2703 genes, respectively. The subtle effects of **TMP195** could be validated for purified T cell (CD3^+^) and B cell (CD19^+^) populations from PHA-stimulated PBMCs. Only CD14^+^ monocytes were transcriptionally sensitive in the context of PHA activation with 443 and 114 genes up- or downregulated, respectively. These findings could be rationalized by the abundance of chemokine and cell-surface genes present in monocytes and the low effect of **TMP195** on lymphocytes. **TMP195** induced mostly upregulation of genes involved in cell proliferation and protein translation.

In 2020 Winter et al. explored the TFMO moiety as antifungal class II HDAC inhibitor. First results showed activity against *Phakopsora pachyrhizi*, the major cause of soybean rust [21]. Further SAR studies with the surrogate *Aspergillus nidulans* led to potent inhibitors **8** and **9** of class II histone deacetylase HdaA and related HOS3-type histone deacetylase HosB (Figure 7). The SAR of the linker highlighted the strong dependence on substitution patterns, with linear, para-like geometries to be favored. The amide cap moiety was found to be variable, suggesting only a weak contribution. Interestingly no modifications of the TFMO group were acceptable, leading to a strong decrease in activity as seen with derivatives of **10** and **11** in Figure 7. This led to an alternative suggestion for the binding mode of the TFMO moiety based on the TFMO-SAR and unpublished co-crystallization data of human HDAC4 together with an TFMO inhibitor. Since the found electron density was not consistent with a closed oxadiazole ring the authors proposed an electrostatic interaction caused by a nucleophilic attack of water on the TFMO moiety resulting in hydration of the C=N bond which in turn could lead to ring opening and facilitate further interactions.

Another antifungal application of TFMO containing compounds was reported by Yang et al. [22]. The design of active compounds was guided by previous TFMO HDACi and diphenyl ether moieties, frequently used in pesticides. Unfortunately, only MD simulations were performed with class II HDAC and **12** containing a TFMO moiety (Figure 7). The most potent compound, **12**, showed significant antifungal activity against *Alternaria solani*, *Botrytis cinerea* and *Sclerotinia sclerotiorum* and was superior to boscalid as positive control. The SAR showed the importance of the TFMO moiety as a replacement with a methyl group led to a significant decrease in affinity. The best compound, **12**, was employed in vivo on tomato plants and cucumber leaves and had comparable effects to the positive control boscalid. Scanning electron microscopy data showed the rupture of fungal mycelial as a consequence of treatment.

El-Awady et al. reported a TFMO containing HDACi with an imidazopydine moiety as a cap group [23]. Compounds containing a TFMO group were selectively active against HDAC5 as frequently demonstrated by this class II selective motive. Compounds **13**, **14** and **15** (Figure 7) exhibited IC_50_ values of 0.31 µM, 0.30 µM and 0.81 µM, respectively and displayed interesting properties in MCF7 cells by de- and increasing class I HDAC gene expression as observed for **13** and **14**, respectively. Further cell experiments showed the TFMO compounds to be only modestly active.

Since TFMO HDACis have gained a lot of attention over the years as selective probes for class IIa HDACs and incorporate a trifluoro group, Turkman et al. decided to utilize TFMO-containing molecules for positron emission tomography (PET) imaging. For this, Turkman et al. incorporated a [^18^F] into the trifluoro methyl group via a novel late-stage modification using bromodifluoromethyl-1,2,4-oxadiazole as precursor [24]. Compound **[^18^F]TMP195** is separatable from the precursor and could be produced with high radiochemical purity (>98%), acceptable radiochemical yield of 3–5% and higher molar activity of 0.33–0.49 GBq/µmol (8.9–13.4 mCi/µmol) when compared to previously reported PET tracers. Unfortunately, **[^18^F]TMP195** exhibited poor aqueous solubility and made in vivo application difficult. To circumvent the poor physiochemical properties of **TMP195**, namely the low affinity of 800 nM in the hands of Turkman et al. and the aqueous solubility, a SAR study was conducted and **16** identified as potent inhibitor with sub nM affinity and improved aqueous solubility (Figure 7) [25]. The SAR study of two linking units showed the 1,4-linker, mimicking a lysine to be superior than the 1,3-linker. A pyridine linker showed improved solubility accompanied with a decrease in potency. Since the cap moiety is most amenable to modifications in HDACis it was designed to be less rigid and lipophilic by incorporating amino acids to mimic the histone surface. Out of the three tested amino acids D-alanine proved to be most active compared to D-serine or rac-alanine together with the benzylic cap moiety. Compound **16** displayed overall higher affinity compared to **TMP195** and was selective against HDAC7 and HDAC9. Evaluation in HT-29 cells confirmed the results and showed superior activity compared to TMP195. Further testing in mice brains demonstrated the ability to permeate the blood–brain barrier (BBB) and accumulate via specific binding of class IIa HDACs as showed with PET and self-blocking experiments, where non-radioactive **16** reduced the uptake in vivo by 47%. Worth pointing out is the crucial sensitivity of the TFMO group to modifications rendering the precursor inactive, which is beneficial as it will not compete with the PET probe.

Intrigued by a report showing to improve the phenotype of Huntington disease (HD) by a HDAC4 genetic suppression in the R6/2 mouse model [26] Stott et al. took interest in targeting class IIa HDACs [27]. Validation of a previously reported potent HDAC4 inhibitor **17** by Hebach et al. [28] (Figure 7) showed a 150-fold selectivity for class IIa in cells, high kinetic solubility, negligible P-gp efflux, high permeability, a short residence time of 2.5 min and poor metabolic stability in mouse liver microsomes. A SAR study of the TFMO moiety confirmed the importance of the CF_3_ group at the C5-position. Replacement with CF_2_H and a CF_3_-C3 regioisomer led to a significant decrease in activity. A SAR study of the cap moiety aimed to improve the metabolic stability led to a replacement of the basic amine with a pyrrolidine group and reduced the overall lipophilicity by a factor of 0.6 leading to **18** (Figure 7). Further investigation showed the stereochemistry to be important and led to a D-alanine analogue **19**, similar to previously undertaken studies. Compound **19** was further studied regarding biochemical and cellular activity. It could be shown that **19** was a potent class IIa inhibitor with a 990- to 2900-fold selectivity against class I and class IIb HDACs in HEK293 cells. Off-target studies at a concentration of 10 µM showed >80% inhibition of muscarinic M1-M5 receptors, 50–80% inhibition of σ1, L-type calcium and sodium channels. A K_d_ value of 3.9 µM was determined for hERG and no inhibition of cytochrome P450 in human liver microsomes. Additional studies showed 100% oral bioavailability, stability in human plasma, blood and gastric fluid with a distribution ration of 3:1 in blood to plasma and a maximum concentration in blood and brain after 30 min but a high clearance of 4.2 L·h^−1^·kg^−1^.

To address the poor 5-year survival of patients with acute myeloid leukemia (AML) and increase treatment success Bollmann et al. were interested in synergistic approaches using a combination of a HDACi and a proteasome inhibitor [29]. Prior studies showed a super-additive effect of the proteasome inhibitor Bortezomib (BTZ) in combination with other active compounds [30]. To reduce the high toxicity of BTZ and omit the low aqueous solubility of **TMP269** a novel class IIa selective HDACi was developed. A simple, three-step synthesis yielded **20** (Figure 7), which exhibited 200-fold stronger cellular HDAC activity, a high selectivity for class IIa over class I/IIb, improved aqueous solubility and a higher isoenzyme selectivity compared to **TMP269** with an inhibitory activity of 114 nM against HDAC4. An analysis via the Chou–Talalay method in four different leukemia cell lines (HL-60, K562, MONO-MAC-6 and THP-1) using a MTT assay showed strong synergistic effects for **20** and **TMP269** with CI values below 0.5. In two of the four cell lines the synergistic effect of **20** was superior to that of **TMP269** with concentrations that retained class IIa preference. Interestingly, **20** is 4–7.7-fold less cytotoxic in leukemia and non-cancer HEK293 cells but still increases caspase activation and apoptosis in a synergistic manner. Compound **20** showed synergistic effects at low concentrations of BTZ while synergistic effects for **TMP269** were only observable at elevated concentrations. Another benefit is the induction of p21 in combination with BTZ compared to **TMP269** plus BTZ and the stronger caspase-mediated apoptosis. Remarkably, **20** had superior effects compared to **TMP269** although both compounds showed similar inhibitory activity in recombinant HDAC enzymes. The authors stated that the general low class IIa HDACi toxicity and more precisely the lower cellular toxicity of **20** together with the synergistic effect could reduce the therapeutic dose of BTZ and result in fewer side effects compared to **TMP269** or pan-HDACi.

### 2.2. 1,3,4-Oxadiazoles

**Figure 8:**
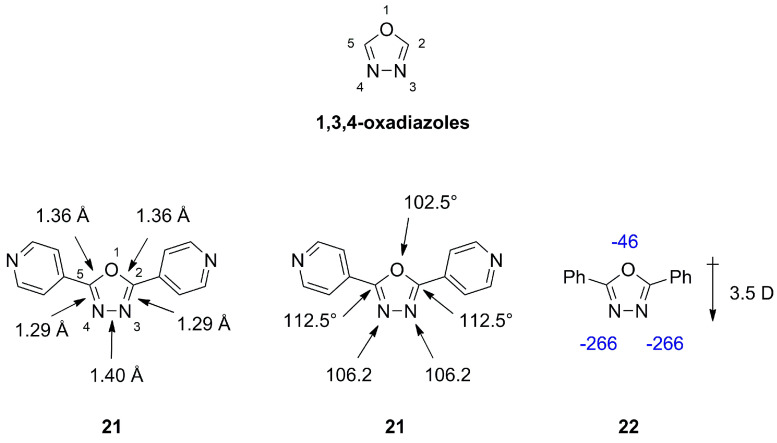
Bond lengths (Å), bond angles (°), dipole moments (D) and calculated hydrogen bond acceptor strengths in kcal/mol indicated in blue for 1,3,4-oxadiazole derivatives.

1,3,4-oxadiazole is a nearly flat and thermally stable heterocycle. It is not fully aromatic, with an index value of 50, compared to furan and thiophen with an index value of 43 and 66, respectively [19]. Bond lengths, angles and dipole moments are depicted in Figure 8 for **21** with a tilt of +3.3° and −3.3° for the pyridine rings. 1,3,4-oxadiazole exhibits bond orders of 1.3124, 1.9062 and 1.3348 for the O-C, C-N and N-N bond respectively [18]. A systematic study conducted by Boström et al. on the interchangeability of 1,2,4- and 1,3,4-oxadiazoles, e.g., **2** and **22**, concluded that the 1,3,4-oxadiazole is superior to the 1,2,4-motive regarding the pharmaceutical properties [2]. It could be rationalized that the roughly two-fold higher dipole moment leads to lower lipophilicity and therefore a higher aqueous solubility, promoting better oral bioavailability, metabolic stability and reduced off target effects and toxicity. Calculations on **22** regarding the hydrogen bond acceptor strength also showed the 1,3,4-moiety to be a more favorable hydrogen bond acceptor [2].

**Figure 9:**
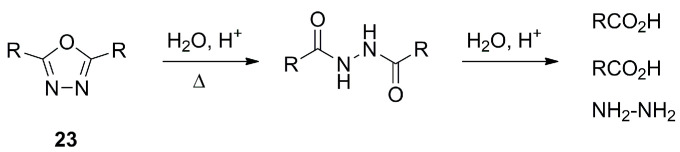
Schematic illustration of the 1,3,4-oxadiazole reactivity.

1,3,4-oxadiazoles like **23** are known to undergo hydrolysis in basic or acidic conditions and to fragment into carboxylic acids and hydrazine (Figure 9) [18]. Electron withdrawing groups at C2 and C5 enhance this reactivity, which proceeds through an attack of the nucleophile on the oxadiazole ring carbon. Disubstituted scaffolds are generally compatible with electrophilic substitution on attached residues. Methyl or unsubstituted oxadiazoles can be metalated by *n*-BuLi and other metalating agents to react with alkylating agents.

The difluoromethyl-1,3,4-oxadiazole (DFMO) moiety was reported in 2017 twice in patents by Lee et al. [31] and Kim et al. [32] as a potent and selective HDAC6 inhibitor moiety and has recently been studied in detail by Cellupica et al. [33] and König et al. [34] and evaluated and applied by Keuler et al. [35], Onishi et al. [36] and Ptacek et al. [37]. HDAC6 is particularly interesting because of its unique substrate scope related to non-oncological conditions. Addressing HDAC6 in a therapeutic manner is accompanied by less severe adverse effects compared to class I HDACs. The great therapeutic window is supported by HDAC6 knock-out (KO) studies in mice, developing normally with no apparent phenotype [38].

**Figure 10:**
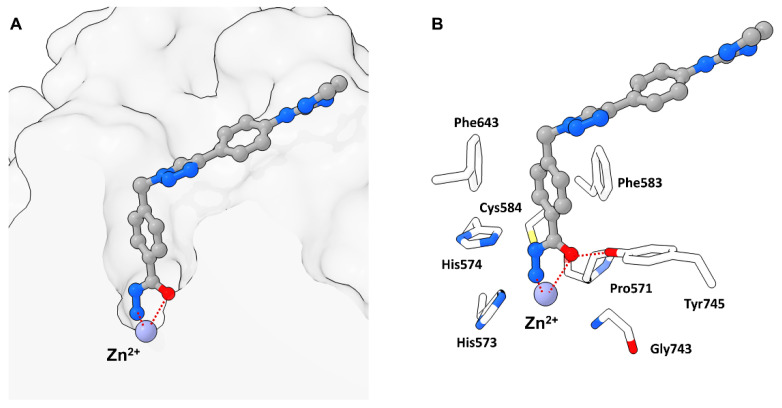
Representation of **26** bound to the class IIb zHDAC6-CD2 (PDB-ID: 8A8Z, 1.60 Å) illustrated using the UCSF ChimeraX visualization software (version ChimeraX-1.4) [16,17]. The inhibitor and the amino acid side chains are displayed in the ball and stick or the stick style and are colored by element or heteroatom, respectively. (**A**) Notable is the smaller binding site compared to class IIa HDACs. Chelating interactions between **26** and the zinc ion are indicated as dotted red lines. (**B**) Visualization of prominent active site residues and interactions with **26** indicated by dotted red lines. Similar to class I HDACs Tyr745 promotes stabilization by interacting with the carbonyl moiety via hydrogen bonding.

In 2022 Cellupica et al. [33] synthesized several DFMO-containing compounds via in silico modelling accompanied by SAR studies and could confirm the unprecedented selectivity and potency of the DFMO moiety against the catalytic domain 2 (CD2) of zHDAC6 [33]. Co-crystallization of **24** and zHDAC6-CD2 resulted in a structure with 1.6 Å resolution (PDB-ID: 8A8Z) and showed no major conformational changes compared to the ligand free structure of zHDAC6-CD2 (PDB-ID: 5EEM). Most interestingly, the electron density in the catalytic site did not match **24** and suggested ring opening of the DFMO moiety. This could be explained by a two-step hydrolytic conversion involving the catalytic activity of HDAC6 with **25** as intermediate, finally yielding a hydrazide as ZBG **26** (Figure 10).

**Figure 11:**
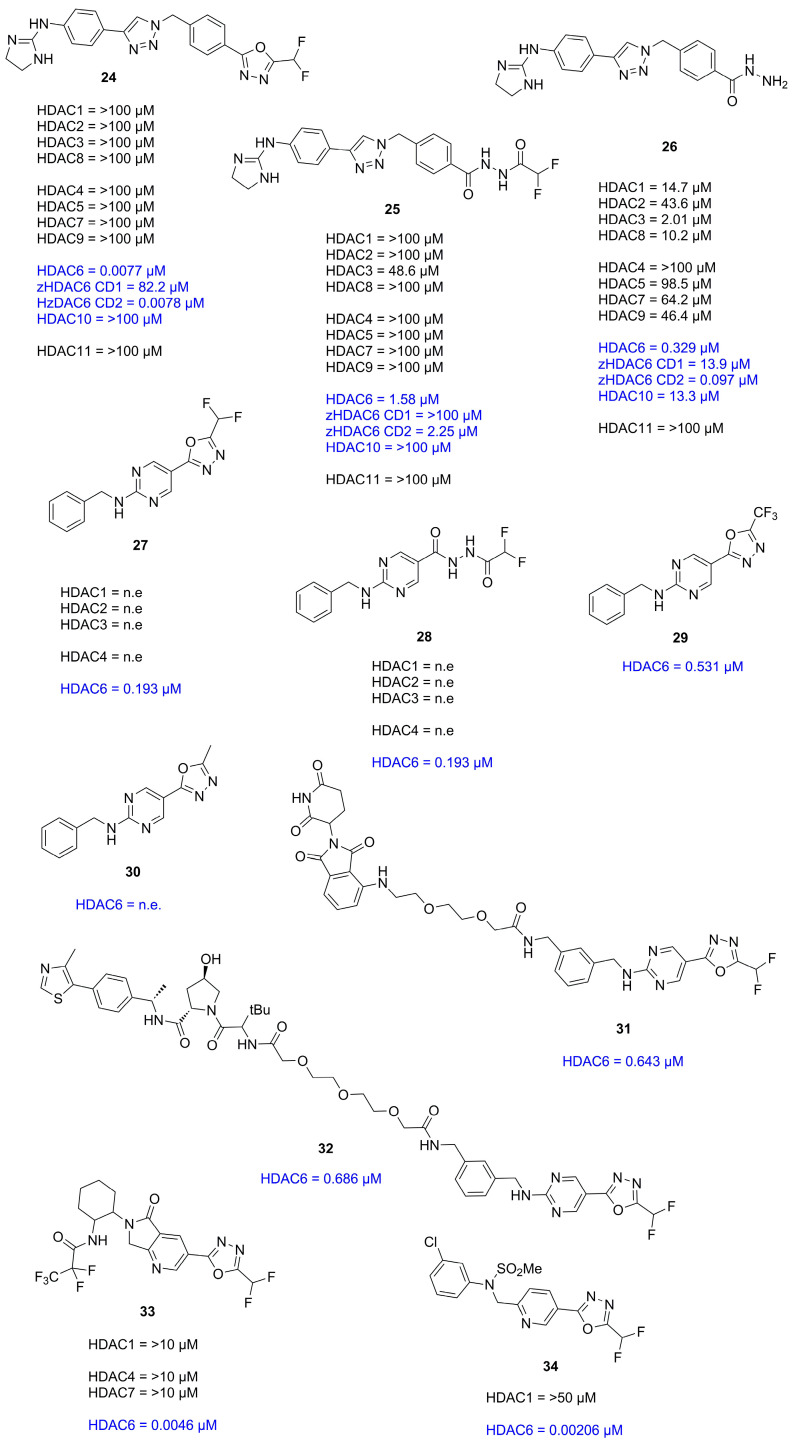
Summary of compounds and their inhibitory activity mentioned Section 2.2. Inhibitory activity against class IIb HDACs is highlighted in blue. Compounds showing no effects against HDAC isozymes were labeled as no effect (n.e).

Modelling suggested an attack of activated water at the DFMO moiety leading to a hydrated intermediate able to engage in hydrogen bonding (HB) with two Histidine residues (His573 and His574) and to preserve interactions with Tyr745. This intermediate could further undergo ring opening and yield an acyl-hydrazide moiety which in turn could serve as substrate for HDAC6 or be hydrolyzed in solution. Conclusively, the hydrazide group would engage in monodentate binding and simultaneously act as a HB donor towards catalytic key residues His573 and His574, whereas the carbonyl oxygen would engage in interactions with Tyr745, acting as HB acceptor. The inherent selectivity towards HDAC6-CD2 was also explained by modelling, suggesting unfavorable geometry in other HDAC isozymes and HDAC6-CD1 for the first hydrolysis step. Further studies of the binding properties revealed a slow binding with an association rate constant of 9.6 × 10^5^ M^−1^ min^−1^ and a calculated residence time of 4.5–5 h. An evaluation of **25** and **26** revealed poorer potency (Figure 11) and selectivity with proceeding hydrolysis and fast-on fast-off kinetics with calculated k_off_ values similar to the first-order constant for hydrolysis of **24**, suggesting the measurement of the actual slow dissociation of the hydrated derivative of **24**. Spin column chromatography coupled with high-resolution mass spectrometry (HRMS) measurements showed the hydrated form of **24** to be the predominant species to coelute with zHDAC6-CD2 and could exclude complexes of **24** and **26** to be the long-lived complex suggesting the in situ formed acyl hydrazide **25** or the closed hydrated intermediate to be the high-affinity species. Supplementary mutation studies involving the residues Y745 and H574, which are known to be involved in stabilization and orientation as well as deprotonation events were performed. The Y745F mutant showed a faster hydrolysis of **24** with one order of magnitude greater dissociation rate constant indicating involvement in the stabilization of the complex. The H574A mutant exhibited a low hydrolysis rate of **24** and **25** implying the hydrolysis reaction to be enzyme catalyzed and the H574 to be involved. Concurrent studies at the Hansen group led to a similar report by König et al. [34] confirming and supplementing findings by Cellupila et al. A fragment-based approach led to heteroaromatic linkers of which a pyrimidinyl derivative performed best. This derivative was complemented with a cap group yielding the full sized HDACi **27** exhibiting an IC_50_ value of 193 nM towards HDAC6 and no activity against HDAC1-4 (Figure 11). Additional synthesis of well-established HDACi and the replacement of their ZBGs with the DFMO moiety led to only moderate active HDAC6 inhibitors, indicating that other factors need to be considered when interchanging ZBGs. A co-crystallization of **27** with zHDAC6 CD2 led to a crystal structure with a resolution of 2.0 Å. An overlay of crystal structures containing **28** and ligand-free zHDAC6 CD2 (PDB-ID: 5EEM) showed a root-mean-square deviation of 0.177 Å indicating no major structural changes. Remarkably, and in agreement with data of Cellupica et al., the DFMO moiety did not match the found electron density thus suggesting a ring opening reaction based on DFMO reactivity. Interestingly and supplementary to data of Cellupica et al., the found electron density matched acylhydrazine **28**, the ring opening product perfectly. The proposed mechanism includes an attack of zinc bound water on the DFMO C2 carbon yielding the ring opened acylhydrazide **28** which is stabilized by many intermolecular interactions. The key interaction is a coordination of the zinc ion by the nitrogen anion with a distance of 2 Å. Further interactions include hydrogen bonds between H574 as well as G743 and a carbonyl group of the acylhydrazine moiety. Additional hydrogen bonds are present between the amide carbonyl and Y745 and the difluoro group and C584 and Y745. A comparison with the structure published by Cellupila et al. showed a shift in orientation of the ZBG by 0.9 Å and a coordination of the zinc ion by the primary amine of the hydrazide moiety. Further differences include a shorter hydrogen bond towards Y745 and the presence of water in the P571 pocket. To shed more light on the binding mechanism the acylhydrazine **28**, the TFMO **29**, and the methyl moiety **30** were synthesized and evaluated. Compounds **28** and **29** showed only weak inhibition of HDAC6 whereas **29** was more potent with an IC_50_ of 531 nM. Further evaluation of binding kinetics was performed with **27** and **29** showing incubation time dependent inhibitory activity (**27**: 5 min–347 nM, 60 min–129 nM; **29**: 5 min–840 nM, 60 min–531 nM). Jump dilution experiments with **27** showed very slow off kinetics which could be confirmed by dialysis experiments indicating a near irreversible inhibition of HDAC6. In contrast, **29** exhibited fast-off kinetics validated in jump dilution experiments, which could be rationalized by a difference in binding mode induced by the sterically more demanding trifluoro group, leading to clashes, thereby destabilizing the binding in the P571 pocket. Further elucidation of the slow binding mechanism suggested “simple slow-binding” mechanism I for **29** and “induced fit” mechanism II for **27**. Essentially, **27** is a slow on inhibitor which serves as substrate analogue with a near irreversible binding mode upon conversion in the active site.

Keuler et al. theorized that chemical knockdown of the entire HDAC6 entity might be superior to simple inhibition of only the catalytic domain (CD2) and developed the first proteolysis-targeting chimeras (PROTACs) with the selective, non-hydroxamate DFMO moiety [35]. Inspired by scaffolds of Yates et al. [39] meta and para-connected HDAC6 ligands with linkers of different polarities were designed for the Von-Hippel–Lindau (VHL) and the E3 ubiquitin cereblon ligase (CRBN). Compounds **31** and **32** (Figure 11) were selected for biological evaluation. The compounds did not impact HDAC1 or HDAC4 levels and did not reduce cell viability. Control experiments with pre-incubation of CRBN and VHL ligands followed by treatment with PROTACs blocked degradation. Similarly, pre-treatment with **Vorinostat** blocked HDAC6 degradation. Non-degrading controls showed no reduction in HDAC6 levels suggesting HDAC6 degradation originates from ternary complex formation.

In search of a therapeutic strategy for tauopathy, Onishi et al. synthesized and evaluated compound **33** (Figure 11) via HTS and medicinal chemistry optimization [36]. As expected on the basis of earlier findings **33** exhibited time dependent inhibitory activity (4.6 nM–60 min) but was highly potent even without preincubation (36 nM). Due to the high selectivity of the DFMO moiety concentrations of up to 10 µM had no obvious impact on HDAC1, 4 or 7. Examination in primary neural mice cells showed concentration dependent acetylation of tubulin without affecting H3 acetylation. In vivo experiments showed that oral administration of **33** led to an increase of tubulin acetylation and plateaued at 1–4 h, gradually returning to control levels after 24 h with values of 1037 ng/mL, 0.25 h, 1722.5 ng·h/mL and 1.76 h for C_max_, T_max_, AUC_0–24 h_ and MRT, respectively. The effect of HDAC6 inhibition was validated using wild type (WT) and HDAC6 KO mice, showing elevated tubulin acetylation in WT but not KO mice. Therapeutic benefits were estimated by administration of **33** for 2 weeks using the P301S tau Tg tauopathy mouse model. A dose of 3 mg/kg in 5-months old mice significantly improved axonal function. The same dose significantly decreased age-dependent tau accumulation in 6- to 9-months-old mice in the FA fraction without affecting the RAB-soluble and RIPA-soluble fractions and led to behavioral improvement regarding the novel object recognition deficit. The authors concluded that **33** can benefit axonal function but that the underlying mechanism of action has not been fully elucidated in part because of HDAC6 versatility and the involvement in various biological pathways.

To evaluate HDAC6 inhibitors Ptacek et al. compared a DFMO containing HDACi side-by-side with common hydroxamate-based HDAC6 inhibitors frequently used in the field [37]. In vitro evaluation showed HDAC10 as primary off-target isoform of hydroxamate-based HDACi while DFMO containing **34** (Figure 11) had outstanding selectivity over all other isoforms. To address cellular potency nanoBRET assays were employed with HEK293T/T17 cells expressing the adequate HDAC isoforms. It was found that potency drops approximately by 100-fold compared to in vitro data. Further evaluation using quantitative Western blotting showed relatively weak correlation between acetylated tubulin and nanoBRET data. Cell toxicity assessment led to the conclusion that in the case of hydroxamate-based HDACi the observed toxicity is correlated to off-target inhibition of class I HDACs. While this is desired in the oncology field it may be unfavorable in chronic diseases. Due to the outstanding selectivity of the DFMO moiety, **34** had less pronounced effects on cell viability at high concentrations making it advantageous in the development of HDACis.

### 2.3. Thiazolidine-2,4-Diones

**Figure 12:**
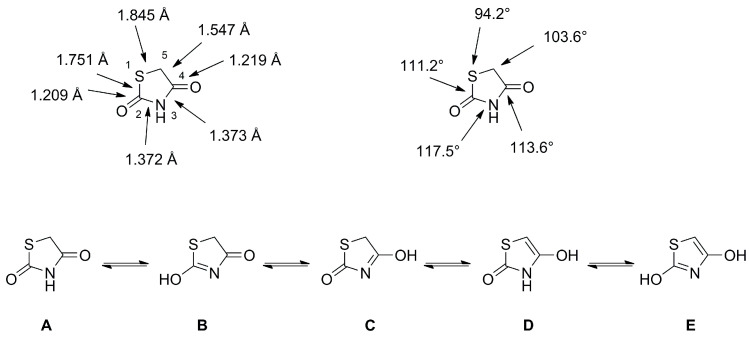
Schematic illustration of bond lengths (Å), bond angles (°) and possible isomeric structures A to E.

Thiazolidine-2,4-diones (TZDs) are five-membered non-aromatic heterocycles which can structurally be derived from thiazole, its aromatic analogue. The TZD moiety can act as a hydrogen bond donor/acceptor and shows no significant deviation from planarity with bond angles and distances indicated in Figure 12 [40]. Despite the possibility of five tautomeric forms, TZDs predominantly exist as diones (A) as high energy barriers disfavor isomerization [40,41,42]. Although the high energy barrier of 24 kcal/mol should suppress keto-enol tautomerization racemization at C5 was observed under physiological conditions [43] and could be rationalized by theoretical studies suggesting a reversible S-oxidation as mechanism [44].

Thiazolidinediones (TZDs) are mainly known for their role in type 2 diabetes, acting as PPARγ agonists thereby decreasing insulin resistance. Elucidation of side effects led to a decline in clinical use and a drop in research publications but the versatile nature of this scaffold promotes the application in many areas including anti-fungal, anti-bacterial and anti-cancer agents and as zinc binding groups [45,46].

**Figure 13:**
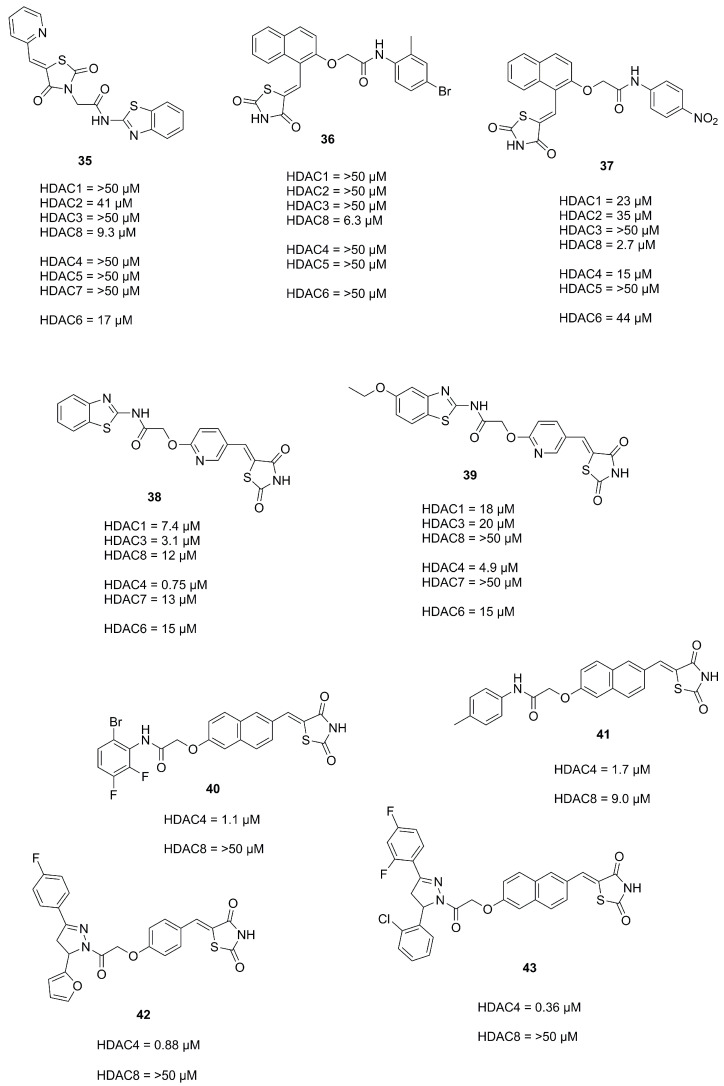
Summary of compounds and their inhibitory activity mentioned in Section 2.3.

In an effort to establish the TZD moiety as a ZBG group Tilekar et al. [47] and Upadhyay et al. [48] synthesized and evaluated TZD containing compounds based on prior studies [49,50] as HDACis yielding compounds **35**, **36** and **37** (Figure 13). Surprisingly, docking studies suggested a binding mode where the TZD scaffold is not participating in zinc binding interactions but is rather part of the linker and cap group [47,48].

Subsequent efforts via a permutation approach yielded a series of overall more elongated compounds with significant increase in activity which were shown to bind to HDAC4 via the TZD scaffold in in silico studies [51]. Docking into the open conformation of HDAC4_o_ (PDB-ID: 2VQJ) resulted in more favorable binding energies than docking into the closed conformation of HDAC4_c_ (PDB-ID: 4CBY). Interactions of the TZD moiety in the active site of HDAC4 composed of complexation of the zinc ion via the C4 carbonyl, a hydrogen bond with His159 via the amide nitrogen and a π-sulphur interaction with Phe168. All tested compounds exhibited a wide range of activity, of which most were two-fold more active on HDAC4, a few were dual inhibitors of HDAC4 and HDAC8 and one compound was selective for HDAC8 but exhibited poor potency. Further optimization identified the benzothiazole moiety as the best cap group and **38** as the most potent compound (Figure 13) towards HDAC4 with an IC_50_ of 0.75 µM and a stabilization of 1 °C in thermal shift assays. Evaluation of **39** against several cancer cell lines showed inhibition of proliferation of hematological (CCRF-CEM) and solid tumor (MDA-MB-231) cells with low impact on non-cancerous HS-27 cells.

Further studies by Tilekar et al. [52] aiming to establish dual inhibitors of HDAC4 and PPARγ to overcome drawbacks of mono-targeting anticancer agents led to a series of compounds with a naphthyl linker and a hydrophobic cap group. The incorporation of the naphthyl linker ensured a shift towards partial PPARγ agonism, which is preferred due to reduced side effects compared to a full agonist and the hydrophobic cap group was installed as an additional requirement for the activity against PPARγ. Potent compounds were further evaluated in dose-response relationships on the basis of a primary screen. A few compounds were found to be dual inhibitors of HDAC4 and HDAC8 but most of the compounds were selective towards HDAC4 with a two-fold higher inhibitory activity. Subsequent optimization of the cap group illustrated compatibility of aromatic, heteroaromatic and heterocyclic aryl groups with significant retention of potency. Again, docking into the open state of HDAC4_o_ (PDB-ID: 2VQJ) with the enlarged pocket resulted in more favorable binding energies compared to closed HDAC4. Docking in HDAC8 showed similar results to prior studies indicating complexation of the zinc ion via the amide carboxyl group. The most potent compound **40** (Figure 13) showed an inhibitory activity of 1.1 µM towards HDAC4 and an EC_50_ of 0.245 µM towards PPARγ. Additional evaluation of **41** in CCRF-CEM cells showed an CC_50_ of 2.8 µM and exhibited 14-fold selectivity against non-cancerous HS-27 cells. Further in vivo studies could show tumor regression in CCRF-CEM mice xenografts.

Similar studies were performed by Upadhyay et al. [53] targeting HDAC4 and VEGFR-2, a vascular endothelial growth factor involved in angiogenesis. To satisfy requirements for the HDAC4 and VEGFR-2 targets the TZD moiety was intended to function as ZBG or to interact with the ATP binding site, and the linker was required for interactions in the binding tunnel of HDACs or to function as a hydrogen bond donor acceptor system, respectively. The cap group is used to establish surface interactions in HDACs or to function as a hydrophobic tail to occupy the allosteric site. A series of compounds was synthesized, and an SAR study conducted. Compound **42** (Figure 13) was most potent with IC_50_ of 0.88 µM against HDAC4 and a remarkable selectivity against HDAC1-3, 7 and 8 but inhibited HDAC6 with an IC_50_ of 7.6 µM. Docking into HDAC4 (PDB-ID: 4CBY) indicated an interaction of the TZD moiety with zinc via the C2 carbonyl, differing to previous docking results suggesting a complexation via the C4 carbonyl. No significant difference was observed upon docking the enantiomer. Evaluation in cancer cell lines MCF-7, K562, A549 and HT-29 showed IC_50_ values of 28.14 µM, 46.27 µM, 19.52 µM and 18.84 µM, respectively. In addition, **42** inhibited in vitro HUVEC (Human Umbilical Vein Endothelial Cell) proliferation (IC_50_ < 10 µM), migration and tube formation and repressed the formation of new capillary in an in vivo CAM assay.

Further studies Upadhyay et al. [54] led to a comparison of linkers by employing a naphthyl and a pyridinyl moiety. Compounds with a naphthyl linker were more potent than their pyridyl equivalent but showed lower stabilization of HDAC4 in thermal shift assays. Compound **43** (Figure 13) was most potent with an IC_50_ of 18 µM, 45 µM, 16 µM, 0.36 µM, 15 µM, 6.3 µM and >50 µM against HDAC1-4 and HDAC6-8 but stabilized HDAC4 by only 0.6 °C. Docking into the open and closed conformation of HDAC4 (PDB-ID: 2VQJ and 4CBY, respectively) showed higher docking scores for the open conformation and higher binding scores for a (S)-enantiomer analogue. Again, the TZD moiety binds the zinc ion with the C4 carbonyl, forms a hydrogen bond to H159 with its amide moiety and is engaged in π-π interactions with H198. Further evaluation of **43** showed an IC_50_ of 2 µM against HUVECs and IC_50_ values of 16.92 µM, 8.92 µM, 17.99 µM and 8.20 µM against MCF-7, K562, A549 and HT-29 cell lines, respectively. Additionally, **43** inhibited endothelial cell proliferation, migration, tube formation and suppressed new capillary formation in growing chick chorioallantois membranes (CAMs). In vivo evaluation in mice xenograft models of human colorectal adenocarcinoma (HT-29) showed a regression in tumor volume by administration of **43** (25 mg/kg) comparable to the reference doxorubicin (20 mg/kg).

**Figure 14:**
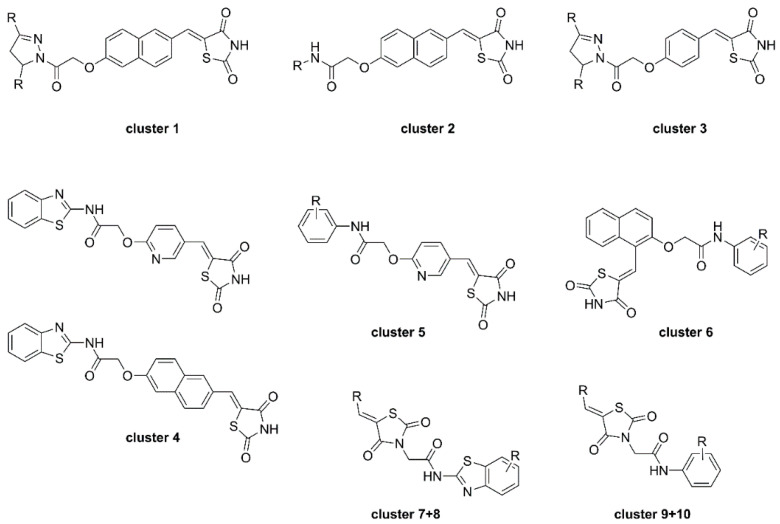
Schematic representation of studied TZD clusters.

To shed further light on the binding mode and kinetic parameters of TZD-containing HDAC4 inhibitors, Schweipert et al. [55] performed a SAR analysis with TZD analogues and several mutants of HDAC4. A total of 225 analogues including previously mentioned TZD inhibitors were evaluated and several docked into the open and closed conformation of HDAC4. First screening revealed 97 compounds with IC_50_ values under 50 µM which were used for further SAR analysis. A similarity mapping with the DataWarrior software yielded ten clusters (Figure 14). Clusters 1–4 were most potent, with IC_50_ values below 2 µM, but differed in linker and cap group with the only common feature being the overall elongated structure and the terminal TZD group, as previously shown in earlier reports. Cluster 5 was structurally comparable to cluster 4 with an overall elongated structure containing a pyridinyl linker but exhibited only moderate activity. Clusters 6–10 consisting of kinked structures or sterically buried TZD groups highlighted the importance of connectivity and steric features showing no potency towards HDAC4. Michaelis–Menten kinetics of clusters 1–4 proved the TZD ligands to be competitive inhibitors which bind in the active site of HDAC4. Additional studies revealed a slow, cap dependent association behavior. Data for clusters 1 and 3 indicated a two-step mechanism with a k_on_ rate constant between 2 and 8 × 10^−3^·s^−1^ and k_off_ values between 0.5 and 1.8 × 10^−3^ s^−1^ resulting in residence times between 9 and 31 min. Data for clusters 2 and 4 pointed towards a one-step binding mechanism with residence times between 8 and 19 min whereas cluster 2 exhibited remarkably slow binding behavior. Evaluation of 25 TZD compounds against 15 HDAC4 mutants verified the medium to strong contribution of the cap group to affinity. Conformational selectivity between the open or closed state of HDAC4 was addressed by docking and experimental findings. Docking suggested energetically more favorable interactions for the open conformation whereas experimental insights pointed towards binding to the closed state. This discrepancy could be rationalized by the strongly shifted equilibrium towards the closed conformation.

### 2.4. Miscellaneous Heterocycles

A broad range of miscellaneous five-membered heterocycles were evaluated by Wang et al. [56] and Liu et al. [57] either as stand-alone ZBGs or in combination with established ZBGs serving as selectivity inducing moieties via foot pocket interactions.

**Figure 15:**
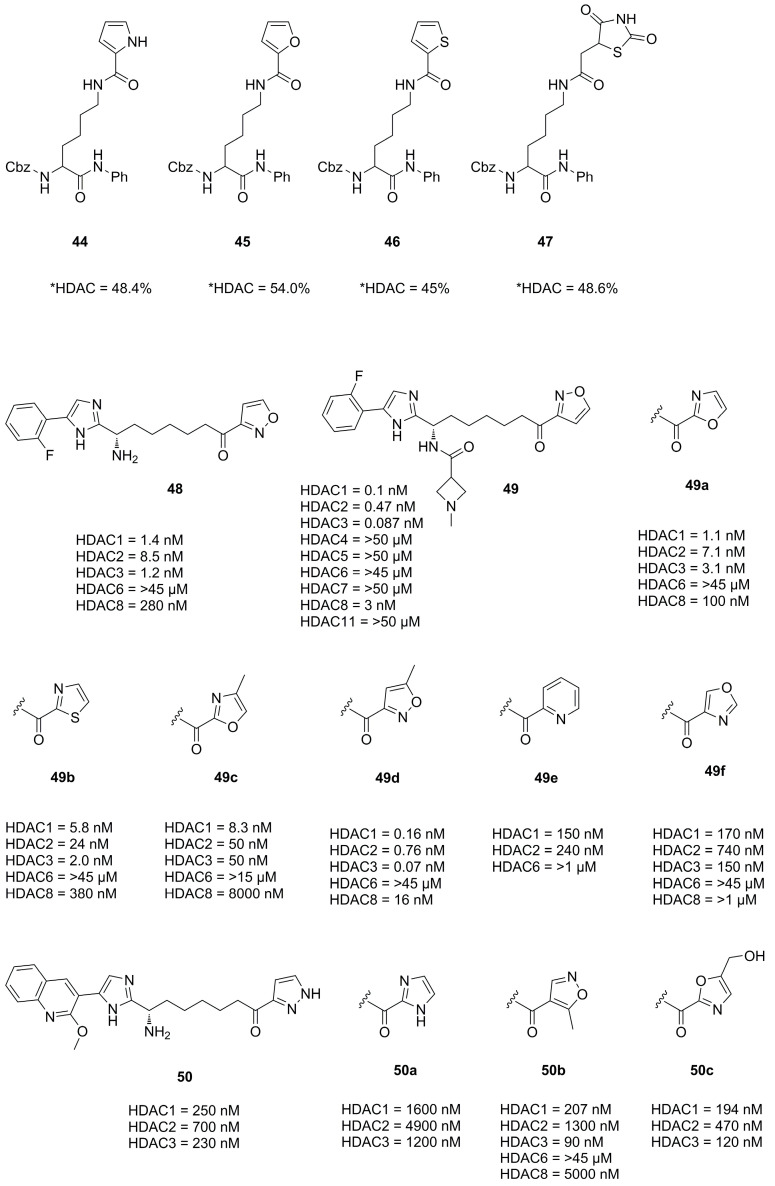
Summary of compounds and their inhibitory activity mentioned in Section 2.4. HDAC* inhibitory activity was measured with HeLa nuclear extract at an inhibitor concentration of 100 µM.

Interestingly Wang et al. synthesized natural substrate analogues and coupled the lysine ε-amine to different heterocycles yielding **44**, **45**, **46** and **47** (Figure 15) [56]. A screening with HeLa nuclear extracts at an inhibitor concentration of 100 µM revealed an inhibitory potential of 48.4%, 54.0%, 45.0% and 48.6% for the mentioned compounds, respectively.

In an effort to develop class I HDAC inhibitors as latency-reversing agents for HIV treatment Lui et al. synthesized a series of compounds with different cap and protruding groups [57]. An SAR study was performed using a ketone zinc binding group attached to an aryl moiety to optimize foot pocket interactions and yielded a series of compounds **48**, **49a**–**f** and **50a**–**f** (Figure 15). The weakest activity was measured in combinations with six-membered aryl rings, which was rationalized by steric demand and clashes in the foot pocket. In contrast, five-membered heterocyclic aryl rings performed significantly better with **49** being the most potent with an IC_50_ value of 0.1 nM. This was even the case for uncompleted compounds like the precursor **48**, lacking a part of the established cap group. Further kinetic assays showed a relatively slow on-rate of 1.82 × 10^−2^·nM^−1^·min^−1^ with a very slow off-rate yielding a residence time of 35 h.

**Figure 16:**
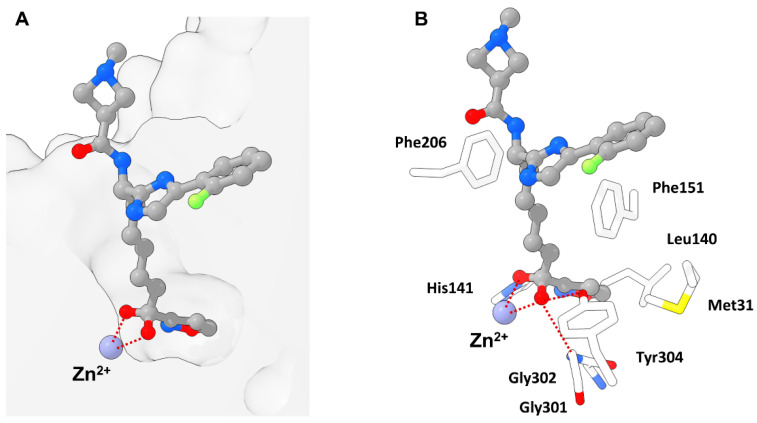
Representation of **49** bound to HDAC2 (PDB-ID: 6XDM, 1.56 Å) illustrated using the UCSF ChimeraX visualization software (version ChimeraX-1.4) [16,17]. The inhibitor and the amino acid side chains are displayed in the ball and stick or the stick style and are colored by element or heteroatom, respectively. (**A**) Notable is the unoccupied and closed acetate release channel also referred to as foot pocket, which is still recognizable as empty volume in the bottom right corner of the clipped surface. (**B**) Visualization of prominent active site residues and interactions with **49** indicated as dotted red lines. The tight fit promotes van der Waals interactions with neighboring residues and hydrogen bonds to Gly301, Tyr304 and His141. The low steric demand of **49** and the gatekeeper function of Leu140 contribute towards a closed acetate release channel and a smaller volume of the foot pocket.

Interactions of **49** and HDAC2 were elucidated by crystallization yielding a 1.56 Å resolution and showing a snug fit inside the foot pocket. The exceptional fit promoted van der Waals interactions with Met31, Gly302 and Leu140 as well as hydrogen bonds to Gly301, Tyr304 and His141 (Figure 16). Interestingly surface interactions with the imidazole moiety composed of hydrogen bonds with the water network on the HDAC2 surface and Asp104 with likewise interactions for the other forked halves of the cap group. Screening against other isoenzymes showed exceptional selectivity and great potency for class I HDACs. Pharmacokinetic evaluation in mouse, rat and dog showed a high volume of distribution and a reasonable half-life with a moderate oral bioavailability of max 18% in mouse. Further evaluation in a Jurkat model using 2C4 cells showed activation of HIV latency with nanomolar potency in the single digit range.

## 3. Cap Group or Linker

### 3.1. 1,2,5-Oxadiazoles

The oxadiazole scaffold and its analogues are frequently used as cap groups or linking units as demonstrated in following studies. Prior to the presented studies, a short introduction on 1,2,5-oxadiazole and its oxo derivative will be given for a more complete understanding of the isoxazole isomers.

**Figure 17:**
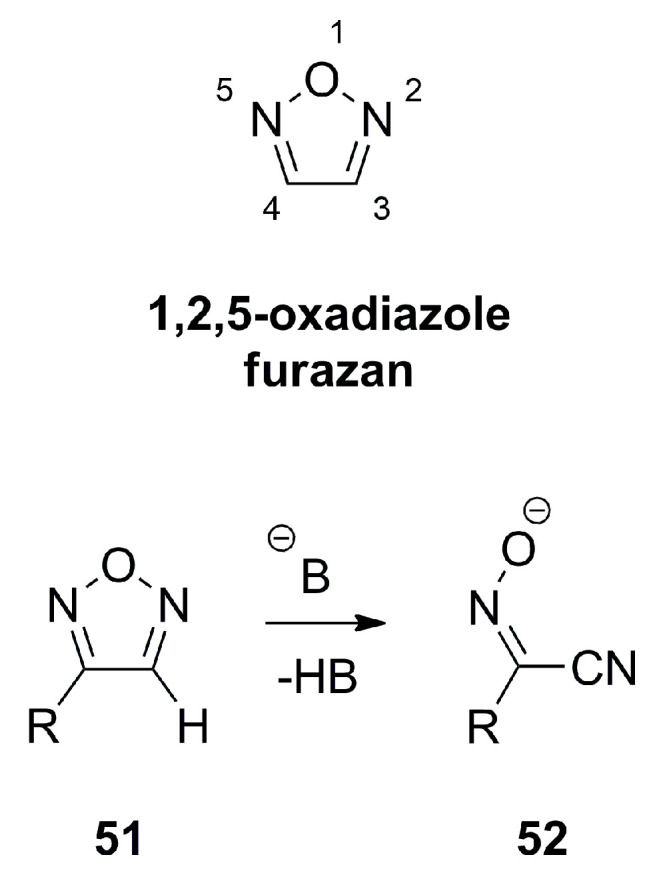
Schematic reactivity of furazans with strong bases.

1,2,5-oxadiazole, also termed furazan is essentially a planar heterocycle which shows single bond character between the N2-O1 and O1-N5 with π-bond orders of 0.32–0.36 and exhibit significant π-electron delocalization for the bonds N2-C3 and C4-N5 with a π-bond order of 0.72–0.82 as well as a bond order of 0.45–0.52 for C3-C4 [18]. Furazans can formally be considered as π-excessive heterocycles with regard to the six electrons distributed over five atoms. Since the π-electron density is mostly located on the heteroatoms, the C-atoms exhibit a π-electron density value of lower than one. Furazans like **51** do not favor tautomerism and react only slowly with nucleophiles despite the low electron density on the C-atoms, but form α-oximinonitriles **52** as ring opening products when treated with strong bases (Figure 17) [18].

**Figure 18:**
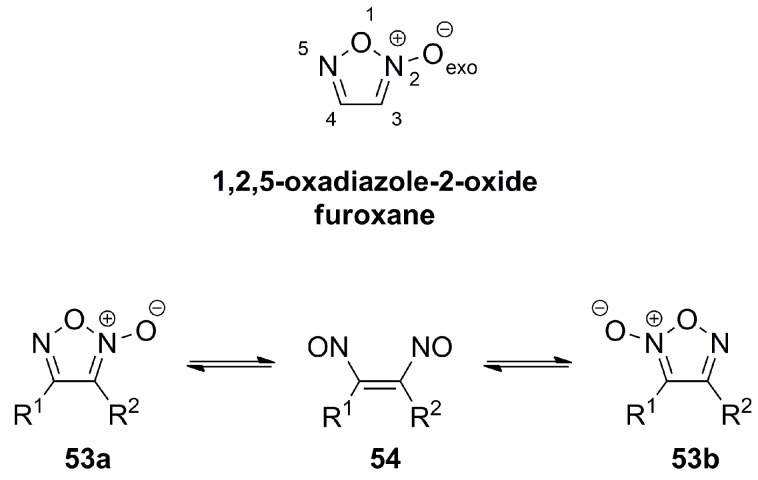
Schematic illustration of the ring chain tautomerism of furoxanes.

The related 1,2,5-oxadiazole-2-oxide, also referred to as furoxane, is also nearly planar, with the exocyclic oxygen projecting by 0.05 Å out of the plane. It has an extended O1-N2 and a shortened N2-O_exo_ bond length. The symmetric distortion propagates extending the N2-C3 and shortening the C4-N5 bond. Consequently, the C3-C4 bond is shortened and exhibits about 30% double bond character. A distinctive feature of furoxanes is the ring-chain tautomerism from **53a** to **53b**, involving dinitroethene **54** as a transition state with an energy of about 120 kJmol^−1^ above that of furoxan (Figure 18) [18].

**Figure 19:**
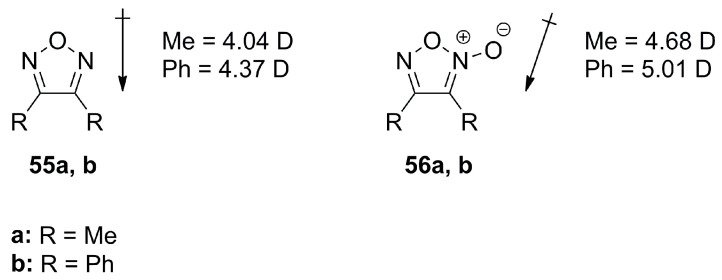
Schematic illustration of differences in dipole moments and vectors between furazans and furoxans.

Furazans **55a**,**b** and furoxanes **56a**,**b** (Figure 19) have a comparably high dipole moment in the range of 4.04–5.01 D with a clear dipole vector shift towards the exocyclic oxygen atom present in furoxanes [18].

**Figure 20:**
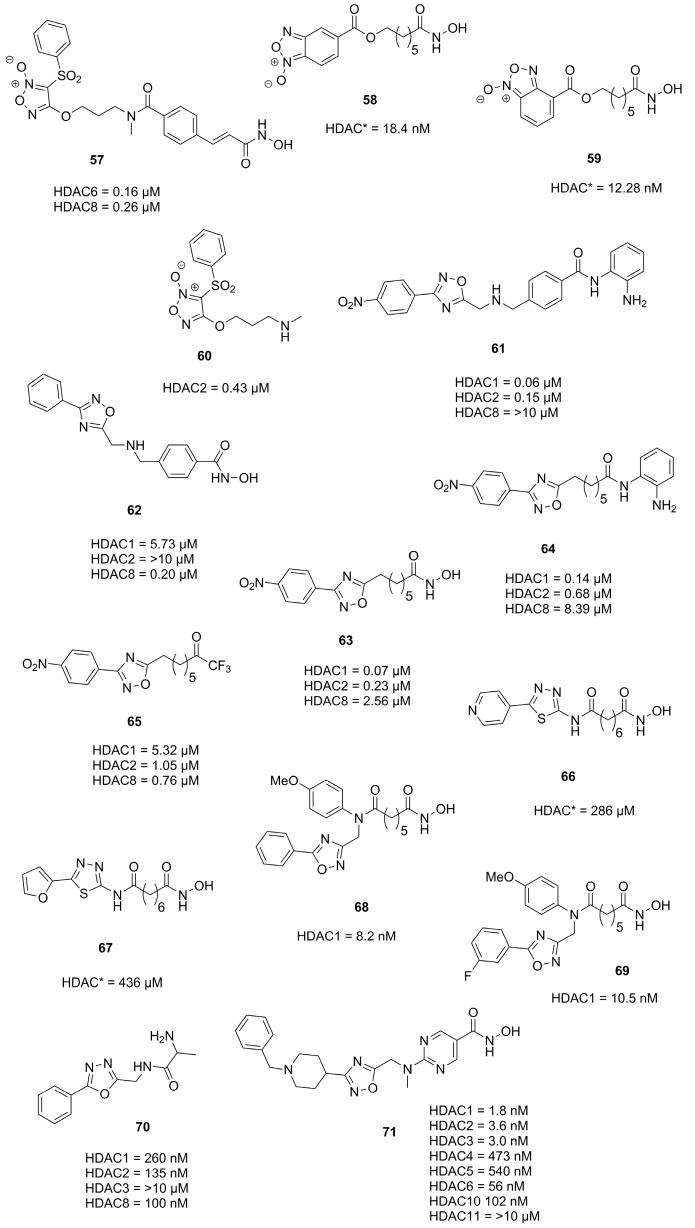
Summary of compounds mentioned in Section 3.1 with their inhibitory activity. HDAC* inhibitory activity was measured using HeLa nuclear extract.

In an effort to design nitric oxide (NO)-donating scaffolds Tu et al. [58] and Duan et al. [59] synthesized and evaluated furoxane containing scaffolds. Compounds **57**, **58** and **59** (Figure 20) were most potent and raised NO levels as observed in the Griess assay. This was most desired since NO is a key signaling molecule involved in regulation of tumor cell proliferation, metastasis, angiogenesis and can modify proteins via S-nitrosylation or Tyr-nitration [60]. Interestingly, the precursor **60** was also active against HDAC2 which was rationalized to originate from S-nitrosylation [61,62]. Evaluation against five cancer cell lines HCT116, SW620, Lovo, MCF-7 and HeLa confirmed the additive effects of the hydroxamic acid motive and the NO donating moiety. Altogether, compound **57** had a better or comparable activity to **SAHA** against HeLa (4.18 µM), HCT116 (3.21 µM), SW620 (4.43 µM), Lovo (5.03 µM) and MCF-7 (7.12 µM) [58]. Compounds **58** and **59** were evaluated against eight cancer cell lines with following IC_50_ values HCT-116 (1.09 µM and 2.73 µM), U937 (12.36 µM and 11.27 µM), B16 (4.97 µM and 30.48 µM), PC-3 (9.18 µM and 14.20 µM), HeLa (9.18 µM and 20.25 µM), HEL (1.26 µM and 3.14 µM), KG1 (0.37 µM and 6.69 µM) and ES-2 (5.07 µM and 44.39 µM), respectively [59].

The following studies employed oxadiazoles described in Section 2.1 and Section 2.2 and analogues thereof. With the intent to replace the carbamate and amide functionalities with oxadiazole as isosteric moiety Cai et al. [63,64] synthesized and evaluated analogues of **Entinostat** [64] and **SAHA** [63]. Selected compounds were evaluated against several cancer cell lines and docked into HDAC2 and HDAC8. Studies on **Entinostat** [64] yielded **61** and **62** (Figure 20) which exhibited better activity against HDAC1 and HDAC2 and complementary activity against HDAC8, respectively. Evaluation of **61** and **62** against several cancer cell lines yielded activities of HCT116 (2.33 µM and 29.3 µM), A549 (6.39 µM and >100 µM), NCI-H661 (4.73 µM and >100 µM), U937 (0.52 µM and 7.21 µM) and MDA-MB-231 (3.18 µM and 14.67 µM). Studies on **SAHA** [63] yielded **63**, **64** and **65** (Figure 20), which showed better or comparable potency against HDAC1, HDAC2 and HDAC8. Again, evaluation in cancer cell lines yielded activity profiles of A549 (5.31 µM, 9.17 µM and 7.68 µM), NCI-H661 (3.09 µM, 0.41 µM and 0.52 µM) and U937 (0.29 µM, 0.46 µM and 0.41 µM) for **63**, **64** and **65**, respectively.

Several other approaches including substitution and optimization strategies yielded compounds **66**, **67**, **68**, **69**, **70** and **71** which are depicted in Figure 20. Guant et al. [65] evaluated **66** and **67** against MDA-MB-231 (4.69 µM and 1.21 µM), K562 (4.15 µM and 1.56 µM) and PC3 (7.75 µM and 3.60 µM) cancer cell lines, respectively. On the basis of previous results Yang et al. [66] replaced a thiophen moiety with oxadiazole yielding **68** and **69**, which performed better than **SAHA** against HDAC1. Evaluation against cancer cell lines yielded following IC_50_ values: HCCLM3 (5.19 µM and 6.56 µM) and HepG2 (1.07 µM and 1.03 µM) for **68** and **69**, respectively. Pidugu et al. [67] designed **70** and evaluated it in a follow up study [68], showing it to inhibit growth of MDA-MB-231 and MCF7 cancer cell lines with IC_50_ values of 0.23 µM and 1 µM, respectively. The compound **71**, synthesized and evaluated by Yang et al. [69], showed potencies ranging between 9.8 and 44.9 nM against 12 cancer cell lines. Application in a Daudi Burkitt’s lymphoma xenograft mice model showed tumor inhibition rates of 53.8% and 46.1% when administered orally with a dose of 20 mg/kg or 10 mg/kg, respectively.

### 3.2. Triazoles

The triazole scaffold is widely applied and popular as the preparation via the copper(I)-catalyzed Azide-Alkyne Cycloaddition (CuAAC) fulfills all criteria for a click reaction: being simple to perform, wide in scope, stereospecific and highly yielding as well as modular with easy separatable non-toxic byproducts. Many research groups applied the CuAAC reaction in the synthesis of compound libraries or as part of optimization strategies which will be presented in the following.

**Figure 21:**
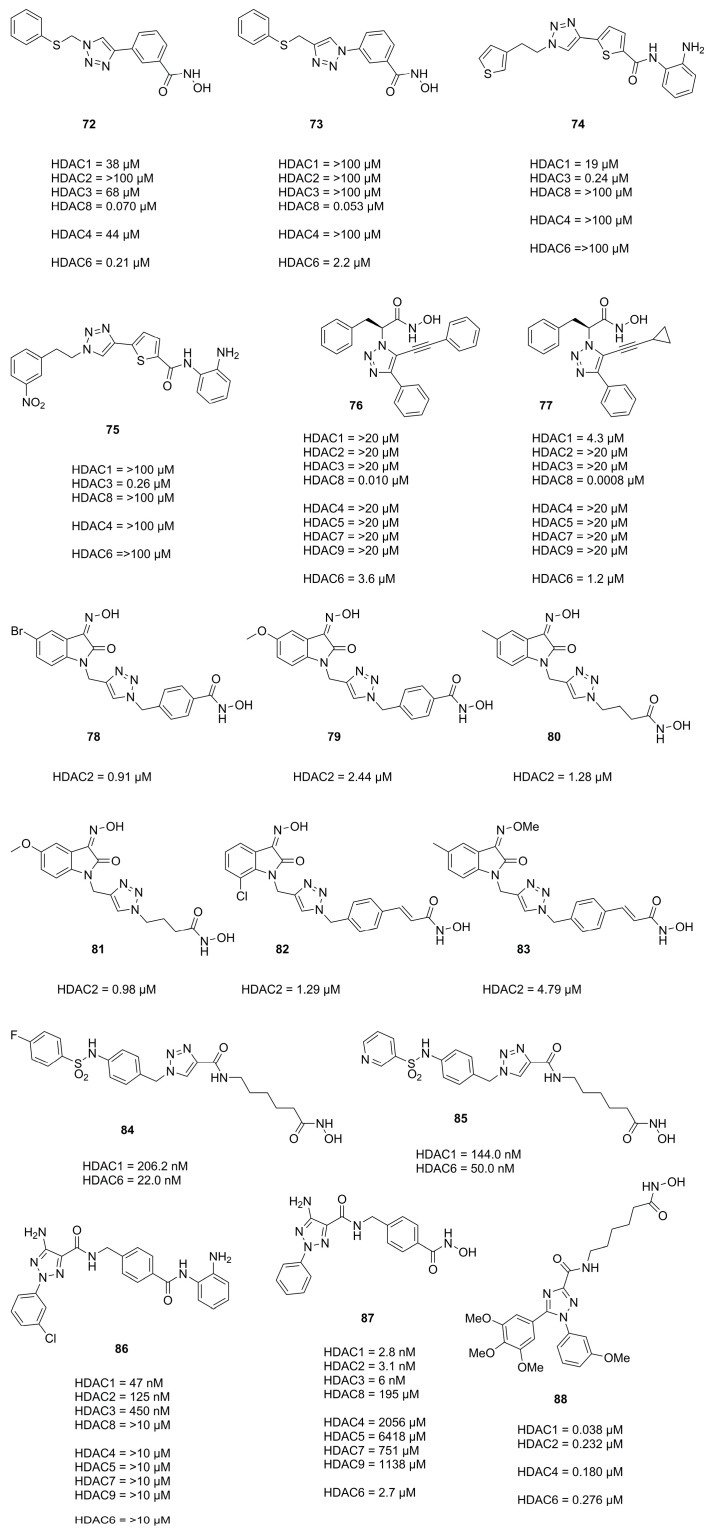
Summary of compounds mentioned in Section 3.2 with their inhibitory activities.

In 2010 Suzuki et al. [70] identified the HDAC8 selective inhibitor **72** (Figure 21) as part of a library prepared using the CuAAC reaction and followed up with further investigation and evaluation identifying the reversed triazole analogue **73** (Figure 21) as a potent and selective HDAC8 inhibitor [71]. Evaluation in four T-cell lymphoma cell lines (Jurkat, HH, MT4 and HUT78) revealed that compound **73**, despite being HDAC8, selectively induced off-target α-tubulin acetylation at a concentration >10 µM and exhibited roughly two-fold higher GI_50_ values compared to **72**. Other studies evaluating a CuAAC synthesized triazole library yielded HDAC3 selective inhibitors **74** and **75** (Figure 21) [72] which exhibited GI_50_ values for HCT116 (1.9 µM and 0.94 µM) and PC3 (1.4 µM and 1.0 µM) cell lines, respectively.

Studies by Ingham et al. identified compound **76** as potent and selective HDAC8 inhibitor and optimized the activity via the CuAAC reaction yielding **77** as best hit (Figure 21) [73]. A SAR study elucidated the features necessary for potent and selective HDAC8 inhibition. These comprised the necessity for a hydroxamic acid and the (S)-configurated phenylalanine moiety. Small aromatic rings directly connected to the triazole moiety and small rings like cyclopropane connected via an alkyne linker were preferred over bulky substituents which showed to significantly reduce the HDACi potency [73].

In search for novel HDACi Huong et al. [74] used the CuAAC reaction to synthesize compounds incorporating the 2-oxoindoline moiety as cap group. Compounds **78** and **79** (Figure 21) were most potent and had comparable activities to **SAHA** with regard to HDAC2 inhibition and potency against four cancer cell lines, respectively. Evaluation against four cancer cell lines showed IC_50_ values of SW620 (8.73 µM and 2.06 µM), PC3 (7.98 µM and 2.62 µM), AsPC-1 (4.18 µM and 1.39 µM) and NCI-H23 (5.46 µM and 1.14 µM) for **78** and **79**, respectively. Follow up studies by Dung et al. [75] yielded compounds **80** and **81** (Figure 21), with potencies better or comparable to **SAHA**, exhibiting IC_50_ values against cancer cell lines of SW620 (0.73 µM and 1.61 µM), PC3 (0.76 µM and 1.74 µM) and AsPC-1 (0.49 µM and 1.49 µM). Further screening and improvements by Dung et al. [76] yielded representatives **82** and **83** (Figure 21). Evaluation against cancer cell lines showed IC_50_ values of SW620 (2.76 µM and 2.71 µM), PC3 (2.17 µM and 2.24 µM), AsPC1-1 (3.03 µM and 1.48 µM) and NCI-H23 (2.89 µM and 0.83 µM).

Mou et al. also used the click reaction to synthesize several triazole scaffolds as potential HDAC6 inhibitors. Evaluation yielded representatives **84** and **85** (Figure 21) as selective and potent HDAC6 inhibitors.

In an effort to improve antitumor activity and reduce adverse side effects Sun et al. designed and synthesized selective and potent HDAC1 inhibitors incorporating a triazole isomer [77]. An SAR study with two different ZBGs, namely hydroxamic acids and aminobenzamides was performed and revealed the superior activity of hydroxamic acids. Notably, the aminobenzamide ZBG was more potent in cells, demonstrating better antiproliferative activity. Different substitutions on position 5 of the triazole moiety showed an amine substitution to be preferred. Aryl substitution on position 2 of the triazole moiety showed no novel, significant trends with a most substitutions yielding comparable potencies. As double substitution also did not improve potency a fused ring system was introduced yielding comparable to more potent activities against HDAC1. Profiling of pharmacokinetic properties in mice (20 mg·kg^−1^) of representative compounds **86** and **87** (Figure 21) yielded K_el_ (0.28·h^−1^ and·0.14·h^−1^), t_1/2_ (2.49 h and 5.03 h), t_max_ (0.42 h and 0.25 h), C_max_ (9558 ng·mL^−1^ and 2715 ng·mL^−1^) and an AUC_0–t_ (15278 h·ng·mL^−1^ and 2917 h·ng·mL^−1^), respectively with an oral bioavailability of 65.1% for **86**. Evaluation in several cancer cell lines showed acetylation of histone H3 and histone H4 in a concentration-dependent manner and a G_0_/G_1_ cell cycle arrest with caspase-dependent apoptosis. Antitumor evaluation of **86** in the MC38 homograft and HCT116 xenografted athymic nude mice inhibited tumor growth and elevated CD4^+^ T cell levels in MC38 homograft. Combinatorial treatment in MC38 mice with **86** and mPD-1 antibody, an immune checkpoint inhibitor led to tumor growth inhibition and complete tumor regression in 5 out of 6 mice, without obvious side effects.

In search of novel HDACi, Aboeldahab et al. [78] synthesized two series of compounds, of which one was bearing a triazole moiety. Representative **88** of series II was not as potent compared to series I compounds but exhibited better antiproliferative activities presumably acting as tubulin inhibitor.

Compound **72** (Figure 21) was used by Marek et al. [79] as probe to explore isozyme selectivity. Marek et al. showed that HDAC8 selective inhibitors adopt a L-shaped conformation allowing them to bind to a HDAC8 specific pocket, mainly formed by the L1 and L6 loop. Soaking experiments of **72** were performed with apo smHDAC8 and the binding mode analyzed. Compound **72** bound in a canonical fashion to the active site with the triazole moiety oriented almost perpendicular to Tyr341 with the phenylthiomethyl cap located in the specific HDAC8 pocket interacting with His292 and Pro291.

### 3.3. Thiazoles

**Figure 22:**
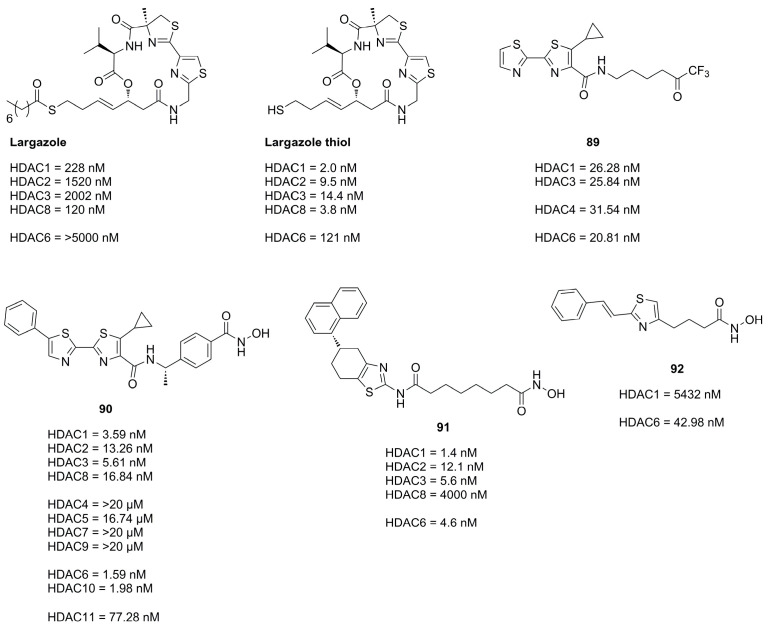
Summary of in Section 3.3 mentioned compounds with their inhibitory activities.

Thiazoles are frequently applied in HDACi scaffolds as cap groups as the bisthiazole motive is part of the well-known depsipeptide **Largazole** (Figure 22), a class I selective HDACi, which interestingly shares the cap core motive with the approved **Romidepsin** HDACi, (Figure 2) [80,81].

Zhang et al. [82] studied the **Largazole** scaffold and could observe that changes in the linker structure could exhibit a significant effect on activity. Introduction of an amide moiety adjacent to the cap group led to inactivity whereas a fluorination adjacent to the cap group was tolerated, leading to comparable class I HDAC inhibitory effects.

A study conducted by Kim et al., shedding light on compatibility of different ZBG in combination with the **Largazole** scaffold, found that the canonical thiol ZBG exhibits the best inhibitory potential. An evaluation of the cap and linker without a ZBG group showed only HDAC8 inhibition of 28% at a concentration of 10 µM.

The application of the bisthiazole cap in HDACi was studied by Gong et al. [83] and Zhang et al. [84] and yielded compounds **89** and **90** (Figure 22). Compound **89**, equipped with a trifluoromethyl ketone ZBG, exhibited the highest activity towards HDAC1-3 and HDAC6 and showed improved antiproliferative activity against cancer cell lines with IC_50_ values of MM1S (0.39 µM), RPMI (0.085 µM), NCI-H929 (1.23 µM), LP1 (0.37 µM), Mino (0.12 µM) and JeKo-1 (0.064 µM) [83]. Compound **90** (Figure 22), equipped with a hydroxamic acid as ZBG showed potent activity towards HDAC isozymes and a high distribution in colon tissue, whereupon it was evaluated in human colorectal carcinoma HT-29 xenograft model, where it was more effective than **Panobinostat** (Figure 2) as control [84]. Compound **90** exhibited a favorable pharmacokinetic profile with T_max_ = 0.67 h, C_max_ = 1.38 ng·mL^−1^, AUC_0–t_ = 924 ng·h·mL^−1^, MRT = 5.22 h, T_1/2_ = 4.23 h and an oral bioavailability of 43% with a dosage of 5 mg·kg^−1^.

Further studies incorporating the thiazole motive were conducted by Sun et al. [77] yielding **91**, which displayed high cytotoxicity in several cancer cell with IC_50_ values better or comparable to **SAHA** and exerted potent antitumor effects in the A-549 zebrafish xenograft model.

Another example is the HDAC6 selective compound **92** which was synthesized by Nam et al. [85] based on previous studies and an SAR evaluation. The authors optimized linker lengths and cap group substituents and concluded based on docking results that cap rigidification was responsible for HDAC6 selectivity allowing the cap group to interact with the small hydrophobic groove of HDAC6.

### 3.4. Imidazoles and Oxazoles

**Figure 23:**
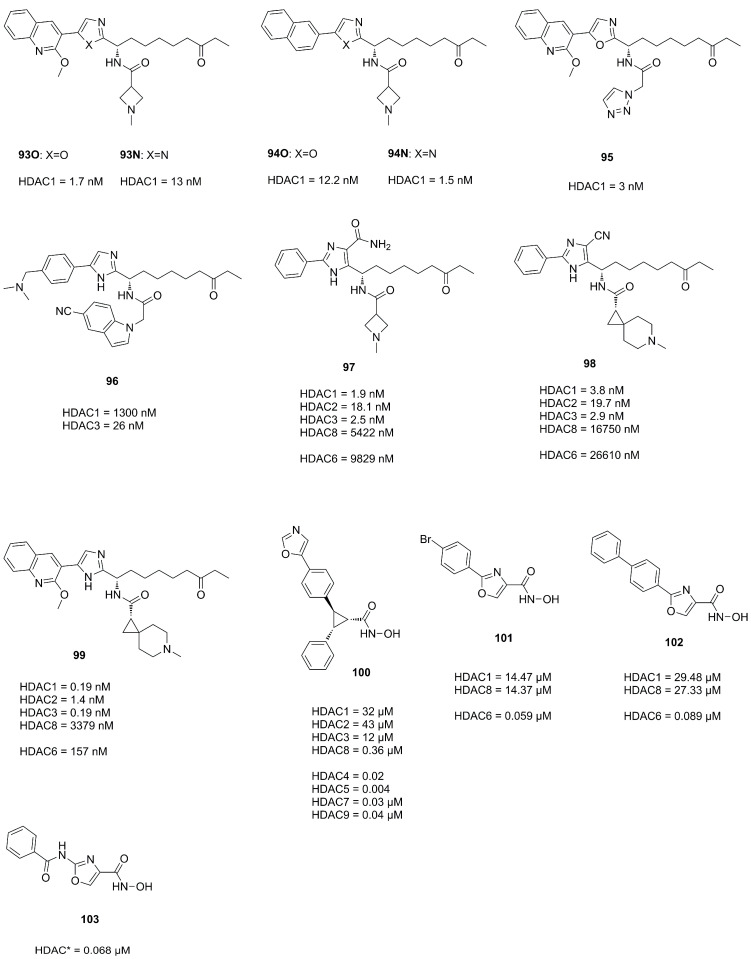
Summary of in chapter 3.4 mentioned compounds with their inhibitory activities. HDAC* inhibitory activity was measured using HeLa nuclear extract.

Based on previous work and SAR studies aiming to target *Plasmodium falciparum* Bresciani et al. [86] identified potent and selective human HDAC class I inhibitors. Interestingly, an interdependence of used cap group and heterocycle was observed. Compound **93O** (Figure 23), with an oxazole moiety, exhibited greater potency in combination with a 2-methoxyquinolin cap, whereas **94N** with an imidazole moiety was more potent in combination with a naphthyl cap. Comparison of pharmacokinetic profiles of an imidazole and oxazole analogue showed improved properties for the oxazole analogue, likely originating from the increased lipophilicity contributing to a better absorption and membrane permeability [86]. Further SAR studies and improvements yielded compound **95**, which had an improved pharmacokinetic profile and a comparable potency to **93O**. Studies focusing on the HDAC3 selectivity yielded compound **96** (Figure 23), which was also synthesized as its oxazole analogue but failed to exhibit HDAC3 activity [86]. Evaluation of the pharmacokinetic profile and in cell studies pointed towards selective HDAC3 inhibition and good stability in plasma and hepatocytes from mice and humans [86].

**Figure 24:**
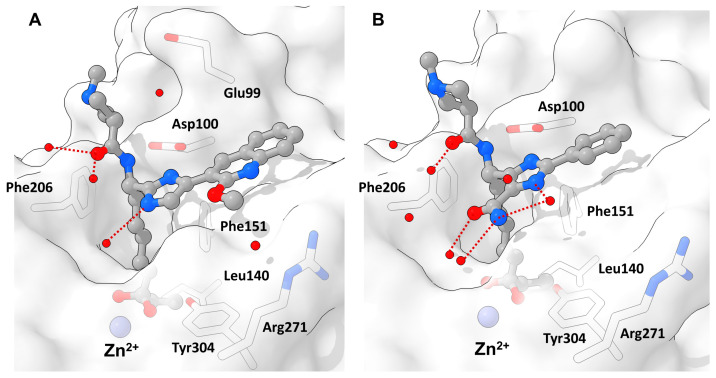
Shown are compounds **93N** ((**A**): PDB-ID: 6WBW, 1.46 Å) and **97** ((**B**): PDB-ID: 6XEB, 1.50 Å) in complex with HDAC2 illustrated using the UCSF ChimeraX visualization software (version ChimeraX-1.4) [16,17]. The inhibitor and the amino acid side chains are displayed in the ball and stick or the stick style and are colored by element or heteroatom, respectively. Ligands are colored dark grey, water is represented as red spheres, interactions are indicated with dotted red lines and amino acids are numbered according to crystal structure. (**A**) **93N** binds in canonical fashion to the active site and engages in interactions with surface bound water. (**B**) The imidazole moiety was flipped and derivatized in **97** to optimize interactions with surface-bound water.

Similar scaffolds were utilized by Clausen et al. [87], Yu et al. [88] and by previously mentioned Liu et al. [57] for HIV treatment studies via a “shock and kill” strategy. The shock is performed by HDAC inhibition reactivating latent HIV reservoirs via gene transcription in resting cells, making them susceptible for “kill” strategies. These studies yielded potent representatives **97**, **98**, **99** (Figure 23) and **49** (Figure 15). Crystal structures elucidated the interactions of the imidazole moiety with a water pocket (Figure 23). In an effort to optimize these interactions, Clausen et al. flipped the imidazole motive and installed additional groups pointing towards the water pocket [87] (Figure 24).

Additional studies incorporating an oxazole moiety were conducted by Bürli et al. [89], Senger et al. [90] and Ahn et al. [91]. Bürli et al. synthesized several compounds in an effort to develop CNS-penetrant class IIa selective HDACi. The authors clearly demonstrated class IIa selectivity and high potency. Evaluation and optimization of ADME properties was challenging and yielded **100** (Figure 23), which had suitable microsomal stability and limited P-gp efflux and could be used as tool compound for proof-of-concept studies in disease models. Senger et al. incorporated an oxazol moiety as linker yielding **101** and **102** (Figure 23). Comparison to thiazole- and oxadiazole analogues validated oxazole as most potent and selective for HDAC6. Ahn et al. synthesized oxazole and thiazole hydroxamate inhibitors and evaluated them in three cancer cell lines. Representative compound **103** had comparable potencies to **SAHA** when tested against HeLa nuclear extract and in three cancer cell lines.

### 3.5. Pyrazoles

**Figure 25:**
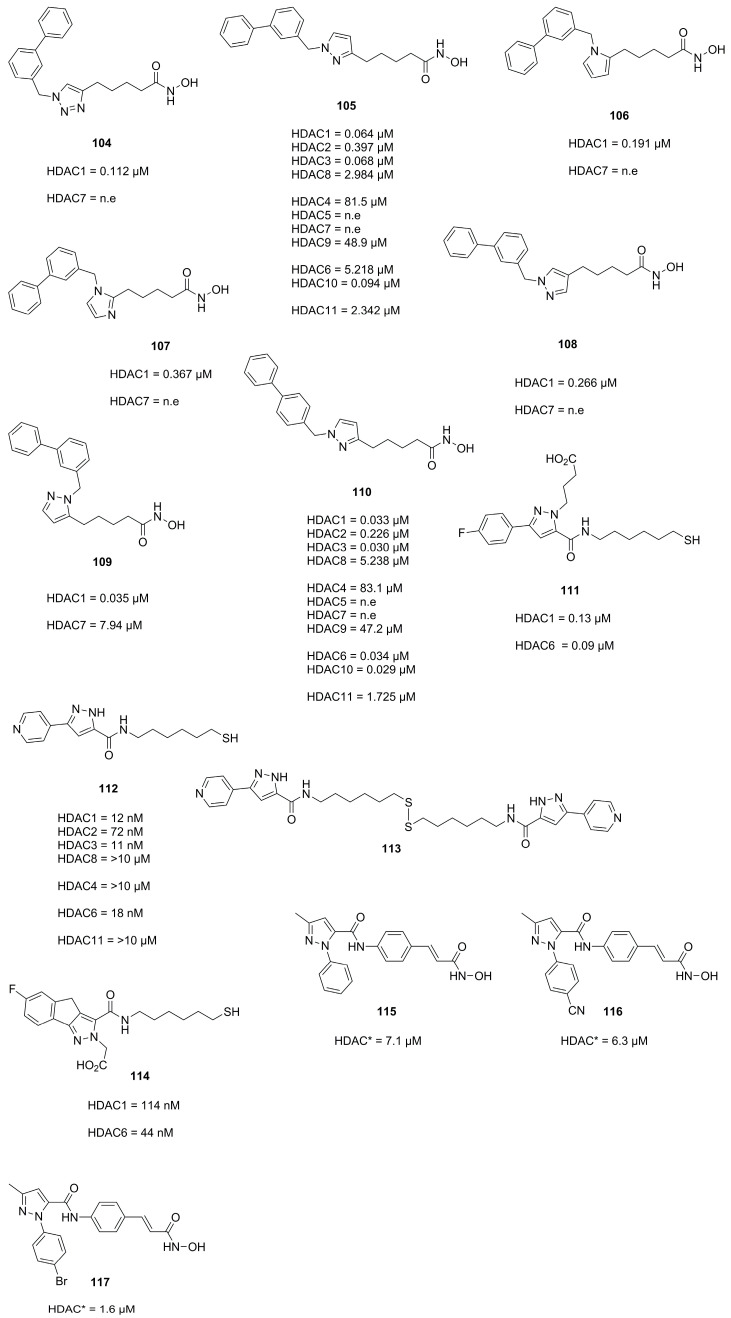
Summary of in Section 3.5 mentioned compounds with their inhibitory activities. HDAC* inhibitory activity was measured using HeLa nuclear extract.

The pyrazole scaffold was used in several publications as part of the cap group (Compounds **104**, **105**, **106**, **107**, **108**, **109**, **110**, **111**, **112**, **113**, **114**, **115**, **116** and **117**, Figure 25). Yao et al. [92] identified the pyrazole moiety via a scaffold-hoping approach starting from **104**. Several heterocyclic scaffolds were synthesized and evaluated. Out of the synthesized scaffolds the pyrazole isomer **105** was the most promising, exhibiting greater potencies than **106**, **107** and **108** and better selectivity than **109** (Figure 25). A SAR study identified compound **110** as most potent, which was evaluated with several other compounds against eight human cancer cell lines yielding GI_50_ values for HeLa (1.23 µM), MCF-7 (1.81 µM), BGC823 (0.26 µM), A549 (0.26 µM), HT1080 (1.33 µM), K562 (0.46 µM), U973 (0.51 µM) and Molt-4 (0.17 µM). [92]

Studies by Wen et al. [93] focused on thiol-based HDACis containing a pyrazole scaffold. Starting from previous work Wen et al. explored the chemical space via nitrogen modifications and regioisomer synthesis which yielded **111** as most potent compound (Figure 25) [93].

Subsequent studies by Wen et al. [94] further optimized the scaffold and evaluated it against seven cancer cell lines. Gained insights of prior studies could be applied to enhance inhibitory activity by modifications at N1 but were omitted due to cell permeability concerns when evaluating the representative **112** (Figure 25) against seven cancer cell lines. Underperformance of **112** in cell assays pointed towards poor stability of the thiol group, which could be circumvented by diol formation. Evaluation of **112** and the diol **113** yielded GI_50_ values for HCT-116 (25.26 µM and 8.93 µM), HT-29 (10.20 µM and 6.92 µM), MCF-7 (12.23 µM and 7.15 µM), MDA-MB-231 (>50 µM and 27.31 µM), A549 (14.43 µM and 6.07 µM), PC-3 (11.03 µM and 4.47 µM) and AsPC-1 (38.85 µM and 25.31 µM), respectively [94].

Further effort by Xu et al. [95] aimed to improve HDAC selectivity by altering the cap motive. Most of the compounds displayed moderate inhibitory activity with **114** being the most selective for HDAC6 (Figure 25) [95].

More research was conducted by Zagni et al. [96] focusing on pyrazole as surface-recognition motive leading to several compounds with inhibitory activity against HDACs and antiproliferative activity against SH-SY6Y tumor cells. It was observed that N1-aryl scaffolds performed significantly better than N1-H scaffolds, with compounds **115**, **116** and **117** showing the most promising activity (Figure 25) [96].

### 3.6. Thiophenes

**Figure 26:**
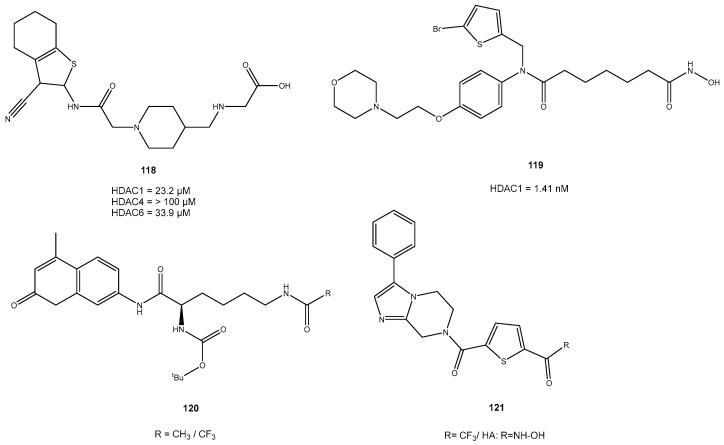
Summary of compounds mentioned in Section 3.6 with their inhibitory activities.

Thiophenes can be utilized as capping groups in HDAC inhibitors. For example, tetrahydrobenzo[*b*]thiophene-3-carbonitriles were used as cap group, developed by Gediya et al. Two different series with this capping unit and a piperidine and piperazine linker were confirmed to be HDAC inhibitors. Compounds with 4(-aminomethyl) piperidine showed good activity against HDAC1 and HDAC6 (IC_50_ value of 23.2 μM and 33.9 μM, respectively). Compounds with piperazine linker, **118** showed comparable values against HDAC6 (IC_50_ value of 13.5 μM). Also, good antiproliferative activity like cell cycle arrest and apoptosis of human cancer cell lines, tested with pro-monocytic human myeloid leukemia (U937) cells and triple-negative breast cancer (MDA-MB-231) cells, could be achieved [97]. Thiophene-based hydroxamate HDACis were also developed to improve their physicochemical properties like solubility. Another reason to introduce heteroaromatic and aliphatic rings such as thiophene is to better fill the cavity of the rim of HDAC1. Yang et al. designed a series of thiophene-based *N*-bis-substituted aromatic amide hydroxamic acid derivatives. Thereby IC_50_ of 1.14 nM for HDAC1 was achieved for one compound, **119** (Figure 26). This molecule was further investigated, and it inhibited the colony formation of the MDA-MB-231 cell line. In general the data demonstrated that all the target compounds showed improved inhibitory activities compared with SAHA [98]. Thiophenes can also be incorporated in the linker unit to receive HDACi as shown by Bottomley et al. Inhibitor-bound and inhibitor-free structures of the histone deacetylase-4 catalytic domain (HDAC4cd) and of an HDAC4cd active site mutant with enhanced enzymatic activity toward acetylated lysines were used to identify histone or protein substrates for class IIa HDACs. Since HDAC4 possesses only slight activity against acetylated lysine containing peptides, an active site mutant (H976Y) was used, which was referred to as the GOF HDAC4. As zinc binding units, both trifluoromethylketone (TFMK) and hydroxamic acid were tested (Figure 26). The hydrolysis of a specific trifluoroacetamide substrate, **120** was inhibited by the mutated HDAC4, whereby IC_50_ values in the nanomolar range were achieved. Even if the two inhibitors 121 only differ in their zinc-chelating groups, a significantly higher potency for the hydroxamic acid was found (IC_50_ of 30 nM for HA and 317 nM for TFMK). IC_50_ values for the WT was much higher with 367 nM for TFMK and 978 nM for HA [99]. 

## 4. Conclusions

Heterocyclic rings, particularly those with five members, are highly valued in the development of active pharmaceutical ingredients. These rings belong to a class of organic compounds that contain at least one non-carbon atom within their structure. The inclusion of heteroatoms such as nitrogen, oxygen and sulfur in the aromatic ring enhances the polarity of the compound and introduces the potential for multiple target-inhibitor interactions. This, in turn, improves the drug’s binding affinity and selectivity at the intended target site. Heteroaromatic rings are considered favorable structures in medicinal chemistry as they can replace common motifs, leading to enhanced metabolic stability, solubility and bioavailability. This review discusses the importance of five-ring heterocycles in HDACis. They occur in three different structural elements of typical HDACis. Five-ring heterocycles can act as ZBG, provide the correct geometry in the binding pocket in the linker part often forming additional hydrophobic or cation-Pi interactions or interact with the protein surface in the head group, which enables the modulation of isozyme selectivity. Of particular interest are five-ring heterocycles that can replace the most commonly used ZBGs in HDACis, such as hydroxamic acid or TFMK. This is significant because these groups potentially mediate non-specific metalloenzyme binding thus increasing the risk for undesired side effects. In addition, hydroxamic acids are suspected of having mutagenic effects and TFMKs are metabolized very rapidly. Five-ring heterocycles can not only replace ZBGs, but also add entirely new beneficial mechanistic properties. The special molecular properties of DFMKs, for example, allows specific cleavage of the oxadiazole ring, presumably by the catalytic machinery of HDAC6, which leads to highly selective HDAC6is with very long residence times. The knowledge on five-membered heterocycles in HDACis summarized here opens up numerous interesting starting points for the future to develop new non-hydroxamic HDACis and provides guidance for medicinal chemists to design and optimize HDACis with improved pharmacokinetic and pharmacodynamic properties.

## Data Availability

Data is contained within the article.

## Abbreviations

ADME	absorption, distribution, metabolism and excretion
AML	acute myeloid leukemia
ATP	adenosine triphosphate
AUC	Area under curve
BBB	blood brain barrier
BTZ	Bortezomib
CAM	chick chorioallantois membranes
CD2	catalytic domain 2
Cmax	maximum serum concentration of drug in a specified compartment of the body
conc.	concentrated
CRBN	E3 ubiquitin cereblon ligase
DFMO	difluoromethyl-1,3,4-oxadiazole
FA	formic acid
GI50	concentration that causes 50% growth inhibition of cells
HB	hydrogen bonding
HD	Huntington disease
HDAC	Histone deacetylase
HDACi	Histone deacetylase inhibitor
HTS	hight throughput screening
IC50	concentration that causes 50% enzyme inhibition
nM	nanomolar
PBMCs	peripheral blood mononuclear cells
PHA	phytohemagglutinin
PROTAC	proteolysis-targeting chimeras
SIRT	Sirtuins
smHDAC	HDAC from Schistosoma mansoni
t1/2	half-life period
TFMO	trifluoro-1,2,4-oxadiazole
Tmax	time of peak plasma concentration
VHL	Von-Huppel–Lindau ligase
ZBG	Zinc binding group
zHDAC6	zebrafish HDAC6
